# A BODIPY-tagged trivalent glycocluster for receptor-targeting fluorescence imaging of live cells†

**DOI:** 10.1039/d4sc08472a

**Published:** 2025-05-27

**Authors:** Chen Guo, Fang-Yu Si, Chen-Han Wang, Ning Wang, Xi-Le Hu, Tony D. James, Jia Li, Chengyun Wang, Xiao-Peng He

**Affiliations:** a Key Laboratory for Advanced Materials and Joint International Research Laboratory of Precision Chemistry and Molecular Engineering, Feringa Nobel Prize Scientist Joint, Research Center, School of Chemistry and Molecular Engineering, East China University of Science and Technology 130 Meilong Rd Shanghai 200237 China xphe@ecust.edu.cn cywang@ecust.edu.cn; b The International Cooperation Laboratory on Signal Transduction, Eastern Hepatobiliary Surgery Hospital, National Center for Liver Cancer Shanghai 200438 China; c National Center for Drug Screening, State Key Laboratory of Drug Research, Shanghai Institute of Materia Medica, Chinese Academy of Sciences Shanghai 201203 China jli@simm.ac.cn; d Department of Chemistry, University of Bath Bath BA2 7AY UK; e School of Chemistry and Chemical Engineering, Henan Normal University Xinxiang 453007 China t.d.james@bath.ac.uk

## Abstract

Multivalent glycoclusters have been extensively used as a targeting agent for drug delivery. However, tools capable of investigating their dynamic interactions with a target receptor remain elusive. Here, we synthesized fluorescently-tagged galactoclusters for the fluorescence imaging of cells that overly express the asialoglycoprotein receptor (ASGPr). A trivalent galactoside was synthesized, to which a boron dipyrromethene (BODIPY) dye was conjugated. The resulting fluorescent glycocluster was used for the targeted fluorescence imaging of liver cancer cells with a high ASGPr expression level. The trivalent probe was also demonstrated to be applicable for super-resolution imaging of ASGPr-mediated ligand endocytosis and the dynamic intracellular translocation to the lysosomes. As such, this study provides a suitable chemical tool for the study of receptor dynamics using fluorescently tagged glycoclusters.

## Abstract 2

We synthesized a trivalent galactosyl BODIPY probe for the super-resolution (STED) imaging of the dynamic endocytic action of asialoglycoprotein receptor (ASGPr).

## Graphical abstract

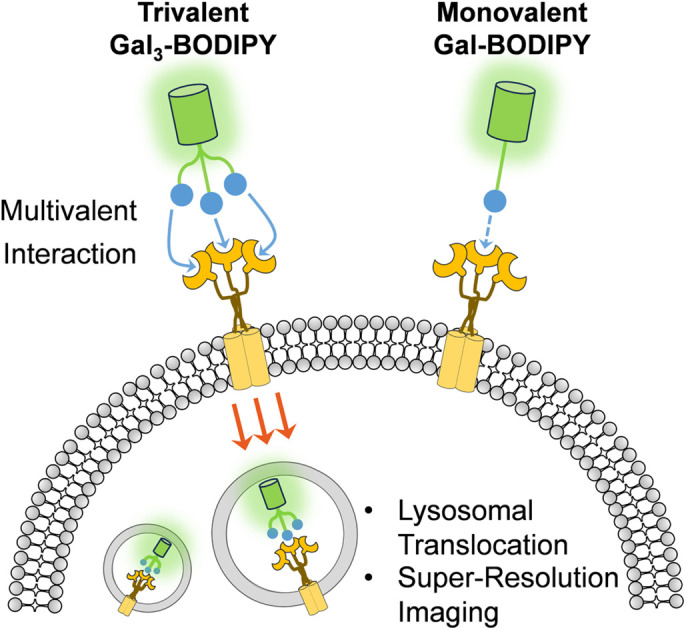

## Introduction

Sugar-receptor interactions are known to mediate a number of biological processes.^1–3^ A representative example is the selective interaction between the asialoglycoprotein receptor (ASGPr) and glycoconjugates bearing galactosyl (Gal) or *N*-acetyl-galactosaminyl (GalNAc) residues, which leads to cell endocytosis.^4^ ASGPr has been determined to be highly expressed in hepatocytes.^5^ Previous studies suggest that ASGPr exhibits important biological functions including disruption of cholesterol metabolism,^6^ alleviation of liver injury^7,8^ and degradation of serum glycoproteins.^9–11^

ASGPr has long been exploited as a molecular target for targeted drug delivery. To enhance the receptor-binding avidity, multivalent glycoclusters have been designed and synthesized. Considering the trimeric nature of ASGPr, a variety of trivalent Gal and GalNAc-based glycoclusters have been developed and used for conjugation with therapeutics for targeted drug delivery.^12^ For example, several GalNAc-conjugated small-interfering RNA (siRNA) therapeutics have been approved by the FDA.^13–15^ Bertozzi *et al.* developed lysosome-targeting chimeras (LYTACs), a novel protein degradation strategy exploiting sugar receptors including ASGPr to mediate endocytosis of membrane-bound proteins.^16^ Furthermore, by targeting ASGPr, small-molecule drugs such as docetaxel^17^ and immuno-virotherapeutics such as oncolytic herpes simplex virus^18^ have been delivered to liver cancer cells in a target-specific manner.

A survey of literature also indicates extensive interest in the development of fluorescent glycoprobes for targeted imaging of live cells and animals. Yan *et al.* synthesized a Gal-conjugated amphiphilic small molecular dye, which can form multivalent nanoparticles in aqueous solution, for NIR-II imaging-guided photothermal therapy (PTT) of liver cancer cells.^19^ Wang *et al.* developed Gal-conjugated fluorescent probes for the targeted detection of Fe^3+^ in the lysosomes of hepatocytes.^20^ Xing *et al.* designed lactosylated fluorescent prodrugs that self-assemble into multivalent nanoparticles for photodynamic therapy and chemotherapy of liver cancer.^21,22^ The same group also constructed aggregation-induced emission (AIE)-active fluorescent nanoparticles bearing multiple copies of glucosamine, mannose or sialic acid for the inhibition of insulin fibrillation.^23^ We have also developed series of fluorescent dye-conjugated glycoprobes^24–26^ and glycoclusters^27–33^ for targeted imaging and therapy of bacterial infection as well as cancer. Despite the rapid development of glycocluster-based delivery systems, fluorescent tools capable of tracking glycocluster–receptor interactions remain elusive.

**Fig. 1:**
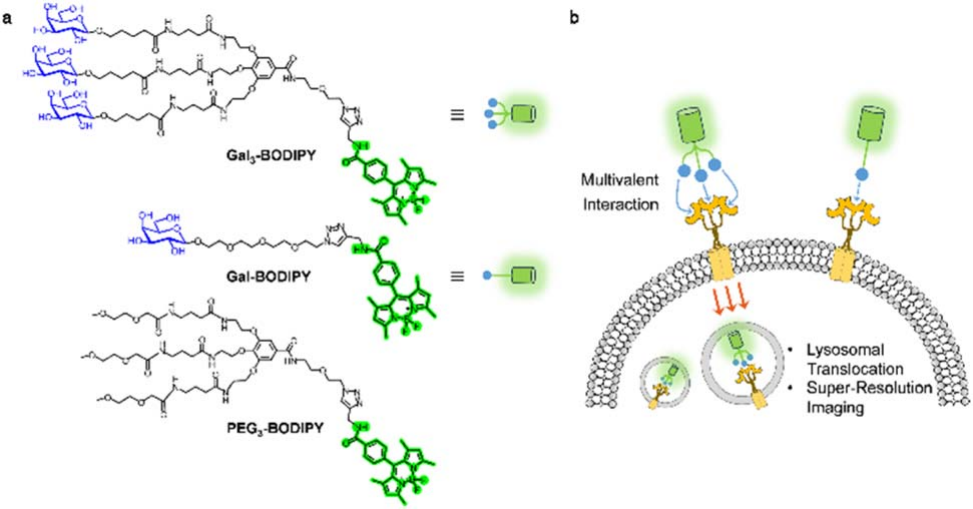
(a) Chemical structures of Gal_3_-BODIPY, Gal-BODIPY and PEG_3_-BODIPY. (b) Schematic illustration of receptor-targeting cell imaging.

Here, we developed boron dipyrromethene (BODIPY) tagged galactoclusters for the multimodal fluorescence imaging of live cells that overly express ASGPr (Fig. 1). Owing to the unique photophysical properties of BODIPY, we were able to achieve the visualization of the glycoclusters upon cell endocytosis and their dynamic translocation to the lysosomes *via* super-resolution imaging techniques. This offers scope for the monitoring of receptor dynamics upon sugar-receptor interactions.

## Results and discussion

The synthesis of the glycocluster is detailed in Schemes S1 and S2.† Gallic acid was used as a template, on which three molecules of galactose (Gal) were displayed. Three *tert*-butoxycarbonyl-protected alkylamines were first introduced to the phenolic positions of gallic acid, and then an azido poly(ethylene glycol) (PEG) was coupled with the carboxylic group of the template through an amidation reaction. After removal of the Boc groups, three pentanoic acid-modified per-*O*-acetyl-1-*O*-β-galactosides were coupled to the template through amide bonds. Finally, reaction with an alkynyl BODIPY derivative *via* Cu(i)-catalyzed azide–alkyne cycloaddition reaction, and removal of the acetyl protecting groups resulted in the desired product Gal_3_-BODIPY being obtained. A monovalent probe (Gal-BODIPY) was synthesized as control with just one galactosyl group introduced to the BODIPY. Another control compound (PEG_3_-BODIPY) where the Gal groups were replaced with methyl groups was also synthesized in a similar manner.

**Fig. 2:**
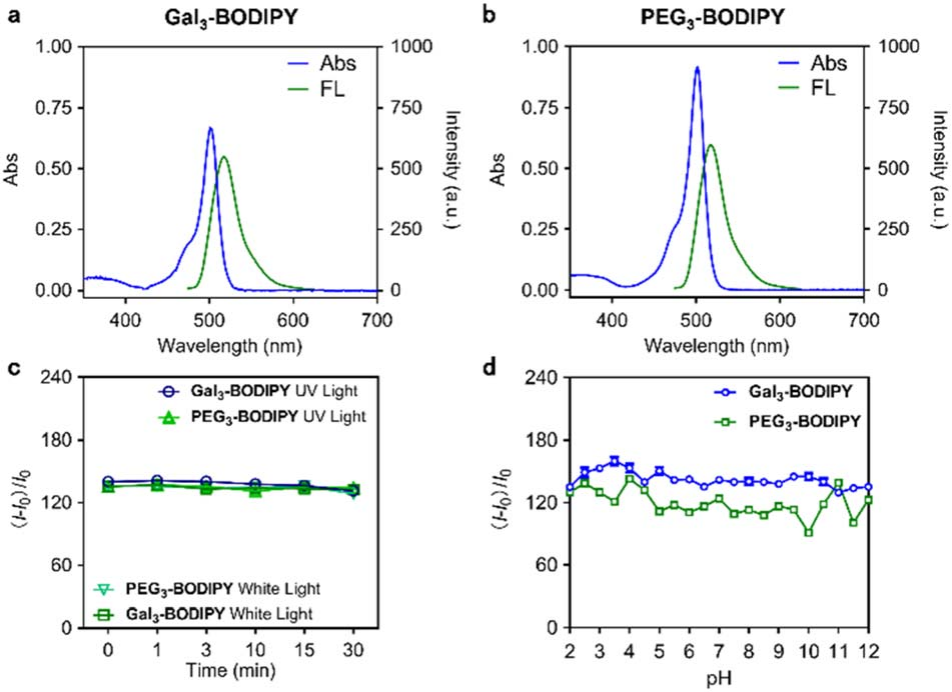
Absorption (40 μM) and fluorescence emission (10 μM, *λ*_ex_ = 488 nm) spectra of (a) Gal_3_-BODIPY and (b) PEG_3_-BODIPY measured in PBS buffer (0.01 M, pH 7.4) (c) fluorescence changes of Gal_3_-BODIPY (10 μM) and PEG_3_-BODIPY (10 μM) in PBS buffer (0.01 M, pH 7.4) under white light (560 nm, 1 W cm^−2^) and UV light (365 nm, 1 W cm^−2^) irradiation with time. (d) Fluorescence changes of Gal_3_-BODIPY (5 μM) and PEG_3_-BODIPY (5 μM) in 21 different pH PBS buffers (0.01 M, pH 2–12). The slit width was set as 5 nm.

With the glycocluster in hand, we determined its photophysical properties. To our delight, the glycocluster is well soluble in phosphate buffered saline (PBS). Therefore, the absorption and fluorescence emission spectra for Gal_3_-BODIPY (Fig. 2a), PEG_3_-BODIPY (Fig. 2b) and Gal-BODIPY (Fig. S1†) were readily obtained. A quantum yield of 0.49 and a lifetime of 1.4 ns was determined for Gal_3_-BODIPY in PBS (Table S1†). In addition, the fluorescence of the glycocluster was not compromised by continuous light irradiation for up to 30 min (Fig. 2c) or exposure to a wide range of pH conditions (Fig. 2d). These results suggest that the BODIPY-tagged glycocluster is suitable for cellular imaging applications.

**Fig. 3:**
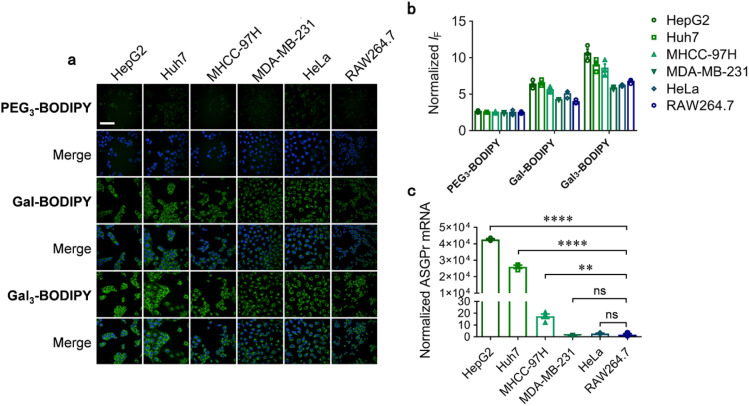
(a) Fluorescence imaging and (b) quantification of HepG2, Huh7, MHCC-97H, MDA-MB-231, HeLa and RAW264.7 cells after treatment with Gal_3_-BODIPY (5 μM), PEG_3_-BODIPY (5 μM) or Gal-BODIPY (5 μM) for 1 h. (c) ASGPr mRNA expression level of the cell lines used for imaging measured by RT qPCR (scale bar = 100 μm).

Next, we turned our attention to evaluate the applicability of the fluorescent glycocluster for targeted cell imaging. Six cell lines including HepG2 (human hepatoma cell), Huh7 (human hepatoma cell), MHCC-97H (human hepatoma cell), MDA-MB-231 (human triple-negative breast cancer cell), HeLa (human cervical cancer cell) and RAW264.7 (mouse macrophage cell) with different ASGPr expression levels were used (HepG2, Huh7, MDA-MB-231, HeLa and RAW264.7 cell lines were purchased from American Type Culture Collection (ATCC), and MHCC-97H cell line was purchased from National Collection of Authenticated Cell Cultures). First the quantitative polymerase chain reaction was first used to determine the relative ASGPr mRNA level of all six cell lines. Then, cells were incubated with Gal_3_-BODIPY, PEG_3_-BODIPY or Gal-BODIPY, and imaged by a high-content screening system. We determined that the fluorescence of Gal_3_-BODIPY was stronger in HepG2 than in other cells (Fig. 3a and b). The quantified fluorescence intensity of the probe agreed with the endogenous ASPGr expression level of the cells (Fig. 3c). Interestingly, the fluorescence intensity of Gal-BODIPY in all the tested cells was seen to be constantly smaller than that of Gal_3_-BODIPY, suggesting a stronger binding between the trivalent ligand and ASGPr. This agrees with the observation in a previous study.^34^ In contrast, a similar level of fluorescence was detected in all six cells for PEG_3_-BODIPY without Gal modification (Fig. 3a and b). We also determined that the fluorescence imaging of HepG2 cells by Gal_3_-BODIPY was concentration (Fig. S2†) and time-dependent (Fig. S2†), and that the glycocluster was not toxic to the cells tested (Fig. S3†). These results help confirm the ASGPr-targeting ability of Gal_3_-BODIPY.

**Fig. 4:**
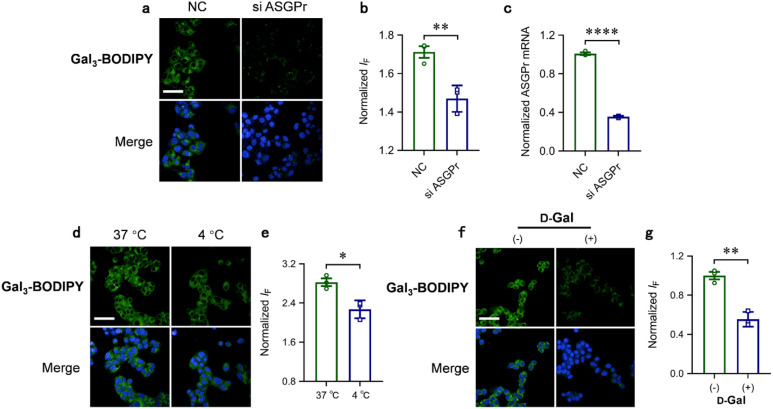
(a) Fluorescence imaging, (b) quantification and (c) mRNA expression level of ASGPr knock-down HepG2 cells and relative control cells. (d) Fluorescence imaging and (e) quantification of Gal_3_-BODIPY cultured HepG2 cells at different temperature. (f) Fluorescence imaging and (g) quantification of HepG2 cells preincubated with free d-galactose (scale bar = 100 μm). BODIPY channel excitation at 488 nm, emission at 500–550 nm. Hoechst 33 342 channel excitation at 405 nm, emission at 435–480 nm.

To corroborate that the targeted imaging is receptor-dependent, several other experiments were carried out. RNA interreference was carried out to suppress the ASGPr expression level in HepG2 cells (Fig. 4c). We determined that cells treated with ASGPr siRNA exhibited a significantly lower cellular uptake of the glycoclusters than those without siRNA treatment (Fig. 4a and b). Incubation of HepG2 cells with Gal_3_-BODIPY at 4 °C significantly decreased the fluorescence intensity with respect to 37 °C incubation (Fig. 4d and e), suggesting the internalization of the glycocluster is kinetically controlled.^35^ In addition, preincubation of the cells with an excess of free Gal suppressed the fluorescence of the glycocluster (Fig. 4f and g).

With promising imaging results obtained, we set out to examine the applicability of the BODIPY-tagged glycocluster for multimodal cell imaging. BODIPY is a class of popular organic dyes often used for live cell imaging because of its high brightness and amenability for super-resolution imaging.^36–39^ We used a Leica STELLARIS 8 STED (stimulated emission depletion) system to image HepG2 cells after incubation with 10 μM of Gal_3_-BODIPY for 5 min under STED and confocal mode (Fig. S4†). With STED, we obtained fluorescence images with suppressed background signals and higher resolution compared to the confocal images. We then set out to explore the super-resolution imaging of ASPGr-mediated endocytic processes using Gal_3_-BODIPY. Ly-Red-BODIPY, a lysosomal tracker developed in our laboratory was used for this experiment (Scheme S3 and Fig. S5†).

**Fig. 5:**
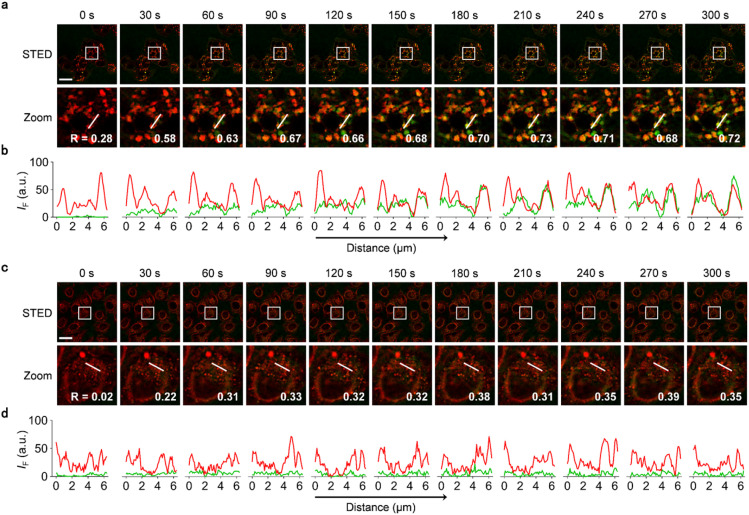
(a) Fluorescence imaging and (b) linear quantification of HepG2 cells incubated with Gal_3_-BODIPY and Ly-Red-BODIPY under STED mode. The Zoom images are those enlarged from the white boxes shown in the STED images. (c) Fluorescence imaging and (d) linear quantification of HeLa cells incubated with Gal_3_-BODIPY and Ly-Red-BODIPY under STED mode. BODIPY channel: excitation at 488 nm, emission at 500–550 nm and depletion with 775 nm STED laser. Ly-Red-BODIPY channel: excitation at 638 nm, emission at 640–700 nm and depletion with 775 nm STED laser. Green and red lines represent the fluorescence intensity of Gal_3_-BODIPY and that of Ly-Red-BODIPY, respectively. The corresponding Pearson's Correlation Coefficient is labelled in the bottom-right (scale bar = 25 μm).

HepG2 and HeLa cells pre-incubated with Ly-Red-BODIPY were treated with Gal_3_-BODIPY and then imaged every 30 s (Fig. 5). During an imaging period of 300 s, we observed that Gal_3_-BODIPY was rapidly internalized by HepG2 cells from 0–30 s and translocated to the lysosomes immediately. Then, the probes resided in the lysosomes over the complete imaging cycle as evidenced by its high Pearson's coefficient values determined when overlapped with Ly-Red-BODIPY (Fig. 5a and b, and ESI movie 1†). In contrast, Gal_3_-BODIPY was hardly internalized by HeLa cells under the same imaging conditions, and a low overlap between the fluorescence of the probe and that of the lysosomal tracker was determined (Fig. 5c, d, and ESI movie 2†). In the meantime, PEG_3_-BODIPY used as a control was found to be barely internalized by both HepG2 and HeLa cell lines (Fig. S6†), which agrees with the results obtained by high-content fluorescence imaging. We also found that Gal_3_-BODIPY was applicable for lifetime imaging, and a lifetime of 3.98 ns was determined for the probe (Fig. S7†). This indicates that BODIPY-modified glycoclusters can be used for lifetime imaging of ASGPr-mediated endocytosis.

## Conclusions

We have synthesized a BODIPY-tagged galactocluster for targeted imaging of live cells. A gallic acid-based tripod was used to display three molecules of Gal, and the resulting trivalent glycocluster was shown to be selectively internalized by cells that overly express ASGPr. In addition, the unique photophysical properties of BODIPY enabled us to visualize ASGPr-mediated endocytosis and intracellular translocation to the lysosomes using super-resolution imaging techniques. This study offers insights for the elaboration of receptor dynamics in live cells using BODIPY-tagged glycoclusters.

## Author contributions

T. D. J., J. L., C.-Y. W. and X.-P. H. designed research; C. G., F.-Y. S., C.-H. W. and N. W. performed research; X.-L. H., T. D. J. and X.-P. H. wrote the paper.

## Conflicts of interest

The authors declare no conflict of interest.

## Supplementary Material

SC-016-D4SC08472A-s001

SC-016-D4SC08472A-s002

SC-016-D4SC08472A-s003

## Data Availability

All data generated during this study have been included as part of the ESI.†^1^H and ^13^C-NMR spectra for unreported compounds can be found in the ESI.†
