# Supplementary material for: A BODIPY-tagged trivalent glycocluster for receptor-targeting fluorescence imaging of live cells
Source: Chem Sci. 2025 May 27;16(25):11481–6. doi: 10.1039/d4sc08472a (PMC12109605; doi:10.1039/d4sc08472a)
Supplement: SC-016-D4SC08472A-s001 [file SC-016-D4SC08472A-s001.pdf]

Supplementary Information (SI) for Chemical Science.
This journal is © The Royal Society of Chemistry 2025

Supporting Information

A BODIPY-tagged trivalent glycocluster for receptor-targeting fluorescence imaging of live cells

Chen Guo,[1,3]† Fang-Yu Si,[1]† Chen-Han Wang,[1,2]† Ning Wang,[1] Xi-Le Hu,[1] Tony D James,[4,5]* Jia Li,[3]* Chengyun Wang[1]* and Xiao-Peng He[1,2]*

[1] Key Laboratory for Advanced Materials and Joint International Research Laboratory of Precision Chemistry and Molecular Engineering, Feringa Nobel Prize Scientist Joint, Research Center, School of Chemistry and Molecular Engineering, East China University of Science and Technology, 130 Meilong Rd, Shanghai 200237, China.

[2] The International Cooperation Laboratory on Signal Transduction, Eastern Hepatobiliary Surgery Hospital, National Center for Liver Cancer, Shanghai 200438, China.

[3] National Center for Drug Screening, State Key Laboratory of Drug Research, Shanghai Institute of Materia Medica, Chinese Academy of Sciences, Shanghai 201203, China.

[4] Department of Chemistry, University of Bath, BathBA2 7AY, U.K

[5] School of Chemistry and Chemical Engineering, Henan Normal University, Xinxiang 453007, China

†Equal contribution

*Email addresses:

t.d.james@bath.ac.uk (T. D. James)

jli@simm.ac.cn (J. Li)

cywang@ecust.edu.cn (C. Wang)

xphe@ecust.edu.cn (X.-P. He)

Content list

S1. Experimental section
S2. Additional figures
S3. Original spectra of new compounds
S4. Additional references

S2. Experimental section

General. All chemicals and reagents were purchased commercially at analytical grade unless otherwise noted. ^{1}H NMR and ^{13}C NMR spectra were recorded on a Bruker AM 400MHz spectrometer with tetramethylsilane as the internal reference. ESI-TOF MS (electrospray ionization-time of flight-mass) spectrometry was carried out on an XEVO G2 TOF system. UV-vis absorption spectra were measured on a Varian Cary 500 UV-vis spectrophotometer. Fluorescence spectra were measured on a Cary Eclipse Fluorescence spectrophotometer.

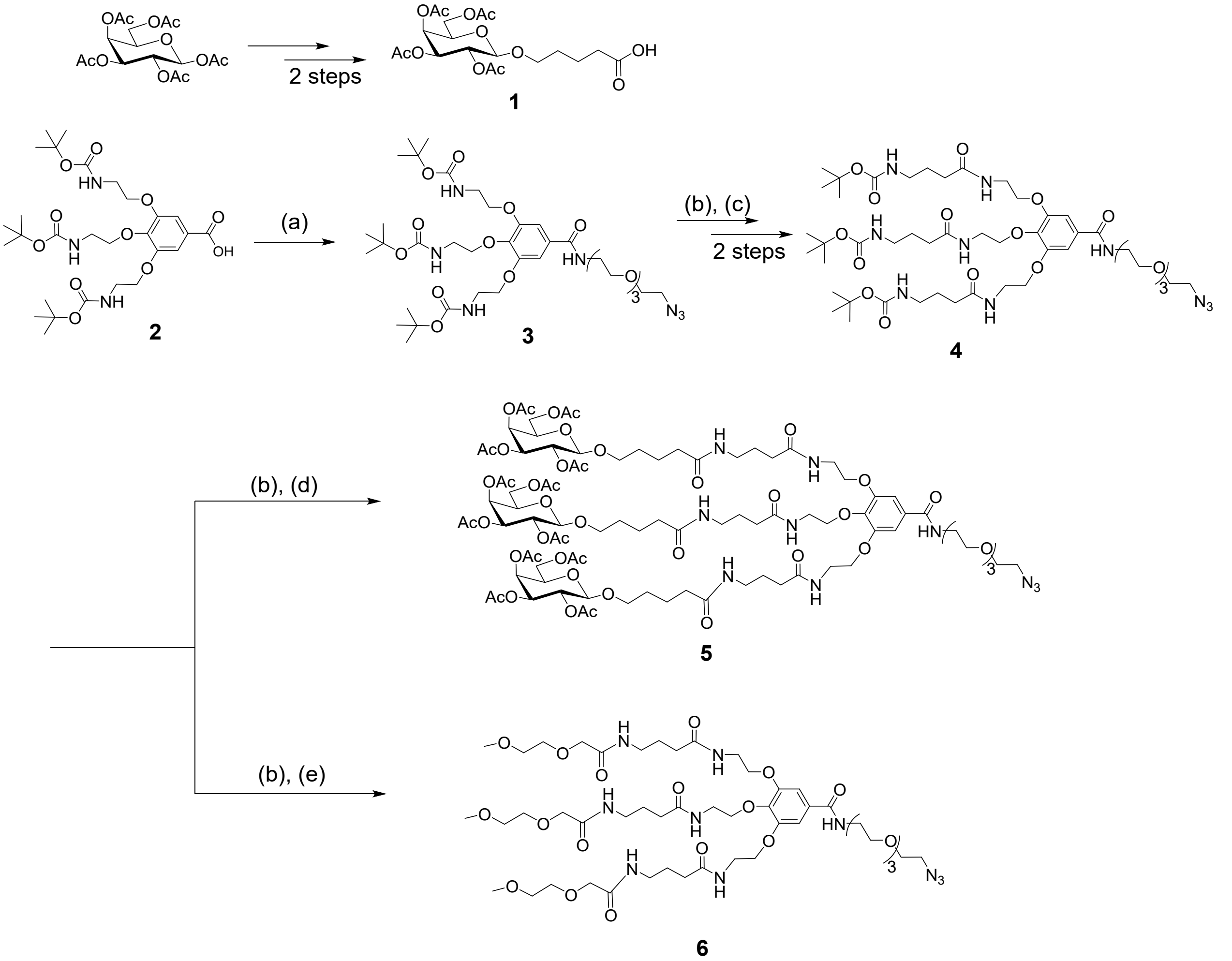

Scheme S1. Reagents and conditions. (a) 2-(2-(2-(2-azidoethoxy)ethoxy)ethoxy)ethylamine, 2-(7-azabenzotriazol-1-yl)-N,N,N',N'-tetramethyluronium hexafluorophosphate (HATU), N,N-Diisopropylethylamine (DIEA), DCM; (b) Trifluoroacetic acid (TFA), DCM; (c) 4-tert-butoxycarbonylaminobutyric acid, HATU, DIEA, DCM; (d) **1**, HATU, DIEA, DCM; (e) 2-(2-methoxyethoxy) acetic acid, HATU, DIEA, DCM.

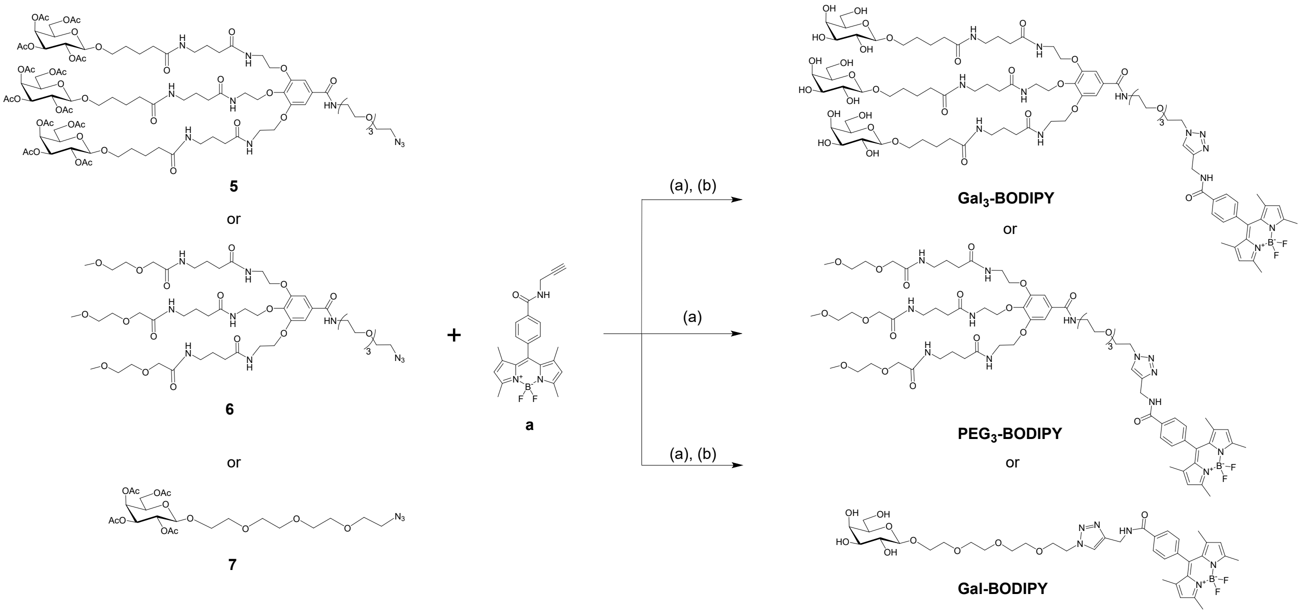

Scheme S2. Reagents and conditions. (a) Sodium ascorbate (VcNa), $CuSO_4·5H_2O$ in DCM/H_2O (5:1, v/v); (b) CH_3OH/CH_3ONa.

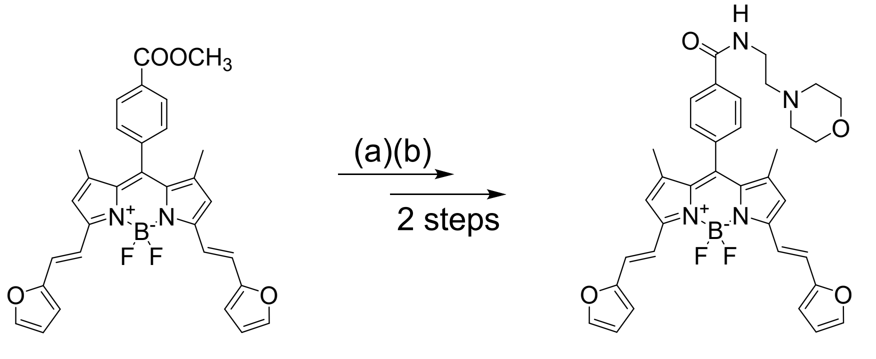

8 **Ly-Red-BODIPY**

Scheme S3. Reagents and conditions. (a) KOH, THF, H_2O at 60 °C; (b) 4-(2-Aminoethyl)m orpholine, HATU, DIEA, DCM.

Synthesis of 3. To a solution of **2** (synthesized according to a previous literature report)[1] (614 mg, 1.02 mmol) in 20 mL DCM was added HATU (467 mg, 1.23 mmol). The resulting mixture was stirred at room temperature for 15 min, and then 2- (2- (2-azidoethoxy) ethoxy) ethylamine (268 mg, 1.23 mmol) and DIEA (395 mg, 3.06 mmol) were added. Stirring was continued for 1 hr at room temperature. Then, the mixture was diluted by DCM and washed by brine. The combined organic layer was dried over dry $MgSO_4$, filtered, and concentrated in vacuum to give a crude product, which was purified by column chromatography to obtain **3** as a colorless liquid (546 mg, 66.6%). R_f = 0.25 (Petroleum Ether (PE)/ Ethyl acetate (EA) = 1:1, v/v). ^1H NMR (400 MHz, $CDCl_3$) δ 7.09 (s, 2H), 6.84 (s, 1H), 5.74 (t, J = 5.4 Hz, 1H), 5.21 (s, 1H), 4.13–4.05 (m, 6H), 3.65 (s, 6H), 3.65–3.59 (m, 9H), 3.58–3.51 (m, 4H), 3.43–3.36 (m, 2H), 3.35–3.31 (m, 2H), 1.47–1.45 (m, 9H), 1.44–1.40 (m, 18H); ^{13}C

NMR (101 MHz, $CDCl_3$) δ 170.4, 166.8(3), 156.0(2), 155.8, 152.3, 130.6, 79.7, 79.6, 72.6, 70.6, 70.6, 70.5, 70.2, 70.2, 70.0, 69.9, 69.8, 69.7, 68.6, 55.6, 50.7, 50.6, 43.5, 40.6, 40.0, 39.3, 38.6, 28.4, 23.2, 18.5, 17.1, 12.6. ESI-TOF MS (*m/z*): $[M + Na]^+$ calcd. for $C_{36}H_{61}N_7O_{13}Na^+$ 822.4220, found 822.4227.

Synthesis of 4. To a solution of **3** (890 mg, 0.55 mmol) in 8 mL DCM, was added 8 mL of TFA. The resulting mixture was stirred at room temperature for 1 hr. Then, the solvent was removed in vacuum to obtain a yellow liquid, which was directly added to a mixture of 4-tert-butoxycarbonylaminobutyric acid (630 mg, 3.10 mmol), HATU (1.41 g, 3.72 mmol) and DIEA (1.2g, 9.30 mmol) in 20 mL DCM. The resulting mixture was stirred at room temperature for 1 hr. Then, the mixture was diluted with DCM and washed by brine. The combined organic layer was dried over dry $MgSO_4$, filtered, and concentrated in vacuum to give a crude product, which was purified by column chromatography to obtain a colorless liquid **4** (816 mg, 75.7%). R_f = 0.38 (DCM/ CH_3OH = 15:1, v/v). ^1H NMR (400 MHz, $CDCl_3$) δ 7.33 (s, 1H), 7.25–7.21 (m, 1H), 7.15 (t, J = 5.5 Hz, 1H), 7.06 (s, 2H), 5.29 (s, 1H), 5.17 (t, J = 6.1 Hz, 2H), 4.10–4.02 (m, 6H), 3.63 (s, 6H), 3.62–3.58 (m, 9H), 3.55–3.50 (m, 2H), 3.33 (t, J = 5.0 Hz, 2H), 3.13–3.04 (m, 6H), 2.29–2.20 (m, 6H), 1.80–1.72 (m, 6H), 1.45–1.40 (m, 4H), 1.38 (s, 27H); ^{13}C NMR (101 MHz, $CDCl_3$) δ 173.4(3), 166.8, 156.5(3), 156.4, 152.1(2), 106.5(3), 79.2(3), 70.6, 70.5, 70.2, 70.0, 69.8, 68.3(2), 50.6, 40.0(3), 39.7(3), 38.9(3), 33.5(3), 29.7(3), 28.4(4), 28.4(3), 26.4. ESI-TOF MS (*m/z*): $[M + Na]^+$ calcd. for $C_{48}H_{82}N_{10}O_{16}Na^+$ 1077.5802, found 1077.5806.

Synthesis of 5. To a solution of **4** (518 mg, 0.49 mmol) in 10 mL of DCM, was added 10 mL of TFA. The resulting mixture was then stirred at room temperature for 1 hr. Then, the solvent was removed in vacuum to obtain a yellow liquid, which was directly added to a mixture of **1** (synthesized according to a previous literature report)[2] (667 mg, 1.48 mmol), HATU (679 mg, 1.78 mmol) and DIEA (576 mg, 4.46 mmol) dissolved in 15 mL DCM. Stirring was continued for 1 hr at room temperature. Then, the mixture was diluted using DCM and washed by brine. The combined organic layer was dried over dry $MgSO_4$, filtered, and concentrated in vacuum to give a crude product, which was purified by column chromatography to obtain a colorless liquid **5** (724 mg, 72.3%). R_f = 0.45 (DCM/CH_3OH = 15:1, v/v). ^1H NMR (400 MHz, $CDCl_3$) δ 7.53 (t, J = 5.4 Hz, 1H), 7.47 (t, J = 5.8 Hz, 2H), 7.33 (t, J = 5.3 Hz, 1H), 7.08 (s, 2H), 6.74 (t, J = 5.7 Hz, 1H), 6.58 (t, J = 5.9 Hz, 2H), 5.37 (s, 1H), 5.36 (s, 1H), 5.18–5.09 (m, 3H), 5.01 (d, J = 3.4 Hz, 2H), 4.98 (d, J = 3.4 Hz, 1H), 4.43 (d, J = 7.9 Hz, 3H), 4.19–4.12 (m, 4H), 4.12–4.09 (m, 6H), 4.08–4.05 (m, 3H), 3.89 (t, J = 7.1 Hz, 6H), 3.63 (d, J = 4.7 Hz, 14H), 3.62–3.60 (m, 4H), 3.53 (d, J = 5.5 Hz, 2H), 3.49–3.44 (m, 3H), 3.34 (t, J = 5.0 Hz, 2H), 3.24–3.18 (m, 6H), 2.46 (s, 6H), 2.28–2.25 (m, 2H), 2.25–2.21 (m, 4H), 2.12 (s, 9H), 2.03 (s, 9H), 2.02 (s, 9H), 1.96 (s, 9H), 1.81–1.75 (m, 6H), 1.68–1.61 (m, 6H), 1.60–1.54 (m, 6H); ^{13}C NMR (151 MHz, $CDCl_3$) δ 173.7(2), 173.6(2), 173.6, 173.5, 170.4(2), 170.2(2), 170.1, 169.8(2), 169.8(2), 167.0, 152.0(2), 139.9, 130.2, 106.2, 101.2(3), 77.4, 77.2, 77.0, 71.7, 70.8(2), 70.6, 70.5(2), 70.5, 70.4(3), 70.1(4), 69.9, 69.8, 69.8(2), 69.7, 68.9, 67.9, 67.9, 67.1(3), 61.2(3), 53.3, 50.6, 41.9(4), 40.0(4), 39.9,

38.9, 38.8, 38.7(2), 38.6, 35.9, 35.9, 33.7, 33.6, 28.8, 28.7, 25.7(2), 25.6, 22.3, 22.3, 20.8, 20.6, 20.6, 20.5(3), 18.3, 13.1. ESI-TOF MS (*m/z*): $[M + Na]^+$ calcd. for $C_{90}H_{136}N_{10}NaO_{43}^+$ 2067.8655, found 2067.8657.

Synthesis of 6. To a solution of **4** (780 mg, 0.74 mmol) in 10 mL of DCM, was added 10 mL of TFA. The resulting mixture was stirred at room temperature for 1 hr. Then, the solvent was removed in vacuum to obtain a yellow liquid, which was directly added to a mixture of 2-(2-methoxyethoxy) acetic acid (300 mg, 2.24 mmol), HATU (1.02 g, 2.68 mmol) and DIEA (868 mg, 6.72 mmol) dissolved in 20 mL DCM. The resulting mixture was stirred at room temperature for 1 hr. Then, the mixture was diluted with DCM and washed by brine. The combined organic layer was dried over dry $MgSO_4$, filtered, and concentrated in vacuum to give a crude product, which was purified by column chromatography to obtain a colorless liquid (614 mg, 75.2%). $R_f = 0.35$ (DCM/CH_3OH = 10:1, v/v). ^1H NMR (400 MHz, $CDCl_3$) δ 7.37 (t, J = 5.4 Hz, 1H), 7.24 (t, J = 5.6 Hz, 5H), 7.17 (t, J = 5.3 Hz, 1H), 7.02 (s, 2H), 4.05 (t, J = 6.1 Hz, 4H), 3.95–3.91 (m, 7H), 3.63 (s, 7H), 3.62–3.60 (m, 9H), 3.53–3.52 (m, 4H), 3.52–3.50 (m, 3H), 3.45–3.41 (m, 5H), 3.37 (s, 6H), 3.36 (s, 3H), 3.32 (d, J = 5.1 Hz, 2H), 3.27 (t, J = 6.3 Hz, 6H), 2.23–2.16 (m, 7H), 2.03–1.98 (m, 4H), 1.84–1.78 (m, 6H); ^{13}C NMR (151 MHz, $CDCl_3$) δ 173.2, 173.2, 170.6, 170.5, 166.9(2), 152.0, 150.3, 140.2(2), 140.0, 130.1, 128.4, 120.2, 106.4, 77.3, 77.1, 76.9, 71.8, 71.5, 71.0, 70.9, 70.6, 70.6, 70.5, 70.3, 70.3, 70.2, 69.9, 69.7, 68.1, 59.0(3), 50.6, 42.0, 40.0, 39.9, 38.9, 38.3, 38.2, 33.6, 33.5, 25.8, 25.7, 18.6, 17.4, 11.9. ESI-TOF MS (*m/z*): $[M + Na]^+$ calcd. for $C_{48}H_{82}N_{10}NaO_{19}^+$ 1125.5650, found 1125.5653.

Synthesis of Gal_3-BODIPY. To a solution of **5** (200 mg, 0.10 mmol) and **a** (synthesized according to a previous literature report)[3] (48 mg, 0.12 mmol) in DCM/H_2O (10 mL/2 mL), were added VcNa (116 mg, 0.59 mmol) and $CuSO_4 \cdot 5H_2O$ (49 mg, 0.20 mmol). The resulting mixture was stirred at room temperature for 12 hr under an argon atmosphere. Then, the mixture was diluted using DCM and washed by brine. The combined organic layer was dried over dry $MgSO_4$, filtered, and concentrated in vacuum to give a crude product, which was purified by column chromatography to obtain a purple-red oily liquid. Then the liquid was re-dissolved in CH_3OH (5 mL), followed by the addition of CH_3ONa (1 M). The mixture was stirred overnight at room temperature, and then Na^+ exchange resin (Amberlite IR 120 H^+) was added to the solution to tune the pH to 7.0. Then, the ion exchange resin was removed by filtration. The organic solvent in the reaction mixture was evaporated, and the resulting residue was purified by column chromatography (DCM/CH_3OH = 10:1, v/v) to obtain **Gal_3-BODIPY** as an oily red-purple liquid (81 mg, 85.0%). $R_f = 0.1$ (DCM/CH_3OH = 5:1, v/v). ^1H NMR (400 MHz, DMSO-d_6) δ 9.20 (t, J = 5.7 Hz, 1H), 8.49 (t, J = 5.7 Hz, 1H), 8.06 (s, 1H), 8.05 (s, 1H), 8.04 (s, 1H), 7.98 (s, 1H), 7.89 (t, J = 5.7 Hz, 1H), 7.77 (t, J = 5.6 Hz, 3H), 7.50 (s, 1H), 7.48 (s, 1H), 7.16 (s, 2H), 6.17 (s, 2H), 4.52 (d, J = 5.6 Hz, 2H), 4.48 (t, J = 5.2 Hz, 2H), 4.03 (d, J = 5.4 Hz, 4H),

3.99 (t, J = 5.6 Hz, 5H), 3.90 (t, J = 6.1 Hz, 4H), 3.81–3.78 (m, 9H), 3.77–3.76 (m, 6H), 3.74–3.70 (m, 6H), 3.70–3.69 (m, 2H), 3.61 (s, 4H), 3.50–3.48 (m, 6H), 3.46 (d, J = 5.8 Hz, 6H), 3.44–3.41 (m, 4H), 3.40–3.36 (m, 6H), 3.30 (t, J = 6.4 Hz, 4H), 3.25 (s, 1H), 3.24 (s, 3H), 3.03–2.97 (m, 7H), 2.44 (s, 6H), 2.10–2.09 (m, 3H), 2.06–2.02 (m, 6H), 1.62–1.58 (m, 5H), 1.53–1.50 (m, 5H), 1.50–1.46 (m, 6H), 1.31 (s, 6H), 1.22 (s, 2H); ^{13}C NMR (101 MHz, DMSO-d_6) δ 172.7(2), 172.6(2), 172.5(2), 165.9(2), 165.9, 155.6(3), 152.2(2), 145.1, 143.1(2), 141.5, 139.9(2), 137.4, 135.1(4), 130.9(3), 129.9, 128.7, 128.5(3), 124.0, 122.0(5), 106.5, 103.9(3), 75.6, 73.9, 71.7(3), 71.0(2), 70.2(3), 70.1, 70.0(4), 69.5(3), 69.2(3), 68.7, 68.6(4), 67.9(3), 60.9(3), 49.7, 38.7, 38.6, 35.6, 35.4(2), 33.3(3), 29.3, 25.9, 22.5, 14.7, 14.6. ESI-TOF MS (*m/z*): $[M + Na]^+$ calcd. for $C_{89}H_{134}BF_2N_{13}O_{32}Na^+$ 1968.9211, found 1968.9170.

Synthesis of PEG$_3$-BODIPY. To a solution of **6** (200 mg, 0.18 mmol) and **a** (88 mg, 0.22 mmol) in DCM/H_2O (10 mL/2 mL), VcNa (215 mg, 1.09 mmol) and $CuSO_4 \cdot 5H_2O$ (90 mg, 0.36 mmol) were added. The mixture was stirred at room temperature for 12 hr under an argon atmosphere. The resulting mixture was diluted with DCM and washed by brine. The combined organic layer was dried over dry $MgSO_4$, filtered, and concentrated in vacuum to give a crude product, which was purified by column chromatography (DCM/CH_3OH = 10:1, v/v) to obtain **PEG$_3$-BODIPY** as a purple-red oily liquid (177 mg, 65.0%). R_f = 0.4 (DCM/CH_3OH = 10:1, v/v). ^{1}H NMR (400 MHz, $CDCl_3$) δ 8.12 (s, 1H), 8.05 (s, 1H), 8.03 (s, 1H), 7.83 (s, 1H), 7.69 (t, J = 5.3 Hz, 1H), 7.56 (t, J = 5.8 Hz, 2H), 7.48 (t, J = 5.7 Hz, 1H), 7.37 (s, 1H), 7.35 (s, 1H), 7.30–7.28 (m, 1H), 7.27 (s, 1H), 7.10 (s, 2H), 5.97 (s, 2H), 4.68 (d, J = 5.4 Hz, 2H), 4.37 (t, J = 5.0 Hz, 2H), 4.08–4.03 (m, 6H), 3.95 (s, 2H), 3.94 (s, 4H), 3.79 (t, J = 5.0 Hz, 2H), 3.66–3.64 (m, 5H), 3.64–3.62 (m, 4H), 3.61 (s, 2H), 3.60 (s, 1H), 3.58 (s, 2H), 3.56 (s, 5H), 3.55–3.54 (m, 3H), 3.54–3.52 (m, 4H), 3.38 (s, 9H), 3.32–3.30 (m, 1H), 3.30–3.26 (m, 5H), 2.54 (s, 6H), 2.29–2.26 (m, 5H), 2.26–2.24 (m, 4H), 2.24 (s, 2H), 1.83 (t, J = 6.9 Hz, 6H), 1.33 (s, 6H); ^{13}C NMR (101 MHz, $CDCl_3$) δ 173.4(2), 170.6(2), 167.0(2), 155.9(2), 152.0, 142.9(3), 140.4(2), 139.8, 138.4(2), 134.5, 131.0(2), 130.1, 128.4, 128.3(2), 121.5(3), 106.2(3), 71.8, 71.5, 70.9, 70.4, 70.3, 70.3(3), 70.1, 69.8, 69.2(4), 68.0, 59.0(3), 50.2(2), 40.0(2), 39.9(2), 38.9(2), 38.3, 38.2, 35.4, 33.6, 33.5, 31.9(3), 30.3, 30.2, 29.7(2), 25.8, 25.7, 14.6. ESI-TOF MS (*m/z*): $[M + H]^+$ calcd. for $C_{71}H_{105}BF_2N_{13}O_{20}^+$ 1508.7654, found 1508.7666.

Synthesis of Gal-BODIPY. To a solution of **7** (synthesized according to a previous literature report)[4] (60 mg, 0.11 mmol) and **a** (53 mg, 0.13 mmol) in DCM/H_2O (10 mL/2 mL), were added VcNa (173 mg, 0.87 mmol) and $CuSO_4 \cdot 5H_2O$ (109 mg, 0.44 mmol). The resulting mixture was stirred at room temperature for 12 hr under an argon atmosphere. Then, the mixture was diluted using DCM and washed by brine. The combined organic layer was dried over dry $MgSO_4$, filtered, and concentrated in vacuum to give a crude product, which was purified by column chromatography to obtain a purple-red oily liquid, which was re-dissolved in CH_3OH (5 mL), followed by the addition of CH_3ONa (1 M). The mixture was stirred overnight at room temperature, and then Na^+ exchange resin (Amberlite IR 120 H^+) was added to the solution to tune the pH to 7.0. Then, the ion exchange resin was removed by

filtration. The organic solvent in the reaction mixture was evaporated, and the resulting residue was purified by column chromatography (DCM/CH_3OH = 10:1, v/v) to obtain **Gal-BODIPY** (34 mg, 85.0%). ^{1}H NMR (400 MHz, DMSO-d_6) δ 9.20 (t, J = 5.7 Hz, 1H), 8.10–8.03 (m, 2H), 8.00 (s, 1H), 7.50 (d, J = 8.2 Hz, 2H), 6.19 (s, 2H), 4.80 (d, J = 4.2 Hz, 1H), 4.69–4.64 (m, 1H), 4.57–4.47 (m, 5H), 4.33 (d, J = 4.5 Hz, 1H), 4.09 (d, J = 6.9 Hz, 1H), 3.87–3.78 (m, 3H), 3.64–3.59 (m, 1H), 3.59–3.46 (m, 13H), 3.29–3.24 (m, 2H), 3.17 (d, J = 4.1 Hz, 1H), 2.45 (s, 6H), 1.33 (s, 6H); ^{13}C NMR (101 MHz, DMSO-d_6) δ 165.8, 155.6, 145.1(2), 143.1, 137.4, 135.2, 130.9(2), 128.7, 128.5, 124.0, 122.0, 104.1, 75.7, 73.9, 70.9, 70.2, 70.1, 70.0, 69.2, 68.6, 68.2, 60.9(2), 49.8, 49.1, 40.6, 40.4, 40.2, 40.0, 39.8, 39.6, 39.4, 35.4, 14.7, 14.6. ESI-TOF MS (*m/z*): $[M + Na]^+$ calcd. for $C_{37}H_{49}O_{10}BF_2N_6Na^+$ 809.3469, found 809.3463.

Synthesis of Ly-Red-Bodipy. To a solution of **8** (synthesized according to a previous literature report)[5] (300 mg, 0.56 mmol) in tetrahydrofuran (15 mL) under an argon atmosphere, was added an aqueous solution of KOH (1 M, 15 mL). The resulting mixture was stirred at 60 °C for 4 hr. Then, the solution was acidified with a hydrochloric acid (1 M) to a pH condition of 2-3 and then extracted three times with DCM. The combined organic layer was dried over dry Na_2SO_4, filtered, and concentrated in vacuum to obtain crude product as a blue solid, which was directly added to a mixture of HATU (215 mg, 0.57 mmol), 4-(2-Aminoethyl) morpholine (75 µL, 0.57 mmol) and DIEA (200 µL, 1.14 mmol) dissolved in 20 mL DCM. The resulting mixture was stirred for 1 hr at room temperature. Then, the mixture was diluted by DCM and washed by brine. The combined organic layer was dried over dry Na_2SO_4, filtered, and concentrated in vacuum to give a crude product, which was purified by column chromatography to obtain a blue solid (220 mg, 91.0%). R_f = 0.35 (DCM/ CH_3OH = 20:1, v/v) ^{1}H NMR (400 MHz, DMSO-d_6) δ 8.62 (t, J = 5.7 Hz, 1H), 8.03 (d, J = 7.8 Hz, 2H), 7.88 (s, 2H), 7.56 (d, J = 7.8 Hz, 2H), 7.45–7.31 (m, 4H), 6.94 (s, 2H), 6.76 (d, J = 3.4 Hz, 2H), 6.69–6.64 (m, 2H), 3.59 (t, J = 4.5 Hz, 4H), 3.48–3.39 (m, 2H), 2.50–2.47 (m, 2H), 2.46–2.41 (m, 4H), 1.39 (s, 6H); ^{13}C NMR (151 MHz, DMSO-d_6) δ 165.5, 152.2(2), 151.6(2), 145.3(2), 141.6(2), 137.3(2), 137.0, 135.0, 132.7(2), 128.6(2), 128.0(2), 123.7(2), 118.4(2), 115.9, 113.8(2), 113.0(2), 66.1(2), 57.3, 53.3(2), 38.2, 14.4(2). ESI-TOF MS (*m/z*): $[M + H]^+$ calcd. for $C_{36}H_{36}BF_2N_4O_4^+$ 637.2798, found 637.2806.

UV-vis absorption spectroscopy. Test solutions of **Gal$_3$-BODIPY** (40 µM), **PEG$_3$-BODIPY** (40 µM) and **Gal-BODIPY** (40 µM) were prepared in a PBS solution (0.01 M, pH 7.4), and the UV-vis absorption spectra of the solutions were obtained on a Varian Cary 500 UV-vis spectrophotometer. The slit width was set at 5 nm.

Fluorescence emission spectroscopy. Test solutions of **Gal$_3$-BODIPY** (10 µM), **PEG$_3$-BODIPY** (10 µM) and **Gal-BODIPY** (10 µM) were prepared in a PBS solution (0.01 M, pH 7.4), and the

fluorescence spectra of the solutions were recorded on a Cary Eclipse Fluorescence spectrophotometer with excitation at 488 nm. For photostability tests, test solutions of **Gal_3-BODIPY** (10 μM), **PEG_3-BODIPY** (10 μM) and **Gal-BODIPY** (10 μM) were prepared in PBS buffer (0.01 M, pH 7.4), and the fluorescence spectra of the solutions were obtained every 3 min for a continuous 30 min upon visible light (560 nm, 1 W cm^{-2}) or ultraviolet light (365 nm, 1 W cm^{-2}) in darkroom. The slit width was set at 5 nm.

Measurement of pH stability. Test solutions of **Gal_3-BODIPY** (5 μM), **PEG_3-BODIPY** (5 μM) and **Gal-BODIPY** (5 μM) were prepared in PBS buffer with varying pH (0.01 M, pH 2–12), and the fluorescence spectra of the solutions were recorded on a fluorescence microplate reader (Varioskan™ LUX) with excitation at 488 nm.

Quantum yield. Quantum yield measurements were done according to a previously described protocol.[6] R6G was used as a reference. The quantum yield of R6G (in EtOH) is 0.98. **Gal_3-BODIPY** was diluted independently from a 5 mM stock solution in DMSO to produce samples in EtOH. Emission spectra were recorded with excitation at wavelengths that overlap with the absorption of the reference dye. The integrals of the resulting emission spectra were plotted as a function of the absorbances at the excitation wavelength for each sample. Quantum yields were calculated from the obtained data according to the following equation.

$$\Phi_{F(\chi)} = \Phi_{F(S)} \frac{A_{(S)}F_{(\chi)}}{A_{(\chi)}F_{(S)}} \left(\frac{n_x}{n_S} \right)^2$$

where Φ_F is the fluorescence quantum yield, A is the absorbance at the excitation wavelength, F is the area under the emission curve, and n is the refractive index of the solvents used. Subscripts S and X refer to the standard and the test samples, respectively. Brightness was calculated to be $\boldsymbol{\Phi}_F \times \varepsilon$.

Fluorescence lifetime. Fluorescence lifetime of **Gal_3-BODIPY** was determined by time-correlated single photon counting using an FLS1000 spectrofluorometer from Edinburgh Instruments. Samples were prepared from a 5 mM stock solution in DMSO at a 1 μM concentration in the respective solvents. Samples were excited using EPL-450 model picosecond pulsed diode laser corresponding to their absorption bands. The resulting fluorescence decays were fitted with both single or double exponential decays. Fluorescence lifetimes of samples were determined from three independent measurements.

Cell culture. HepG2, Huh7, MHCC-97H, MDA-MB-231, HeLa and RAW264.7 cells were cultured in Dulbecco's Modified Eagle's Medium supplemented with 10% fetal bovine serum in a humidified atmosphere of 5% CO_2 and 95% air at 37 °C. Cells were split every two days.

Cell viability. Cells were seeded on clear bottom 96-well plates (Corning, 3599) at different densities. Cells were incubated with different concentrations of **Gal_3-BODIPY** and **PEG_3-BODIPY** for 24 hr. Then, a solution of CCK8 (10 μL/well) was added to each well. After incubation at 37 °C under 5% CO_2 for 3 hr, the absorbance of the solution mixture was measured at 450 nm using a M5 microplate reader. The optical density of the result was directly proportional to the number of viable cells.

Cell imaging. HepG2 (3×10^4 cells per well), HeLa (1.5×10^4 cells per well), Huh7 (1.5×10^4 cells per well), MHCC-97H (3×10^4 cells per well), MDA-MB-231 (1×10^4 cells per well) and RAW264.7 cells (3.5×10^4 cells per well) were seeded in black 96-well microplate with optically clear bottom and cultured overnight. All cells were incubated with different concentration **Gal_3-BODIPY, PEG_3-BODIPY** and **Gal-BODIPY** at different time points, and then Hoechst (1:1000 diluted by PBS) was incubated with cells for 10 min. After rinsing with warm PBS, the fluorescence images were recorded by Opera Phenix high content imaging system and quantified by Columbus analysis system (PerkinElmer, US). BODIPY channel: excitation at 488 nm, emission at 500-550 nm. Hoechst 33342 channel: excitation at 405 nm, emission at 435-480 nm.

Competition assay. HepG2 (2.5×10^4 cells per well) were seeded in a black 96-well microplate with optically clear bottom overnight. Then cells were incubated with 40 mM D-galactose at 37 °C for 2 hr followed by incubation with **Gal_3-BODIPY** (5 μM) at 37 °C for another 1 hr. Then, cells were treated with Hoechst (1:1000 diluted by PBS) for 10 min, and fluorescence images were finally recorded using an Opera Phenix high content imaging system (Perkinelmer, US) and quantified by Columbus analysis system (Perkinelmer, US). BODIPY channel: excitation at 488 nm, emission at 500-550 nm. Hoechst 33342 channel: excitation at 405 nm, emission at 435-480 nm.

STED and fluorescence lifetime imaging. Both STED and fluorescence lifetime images were acquired on a Leica STELLARIS 8 STED microscope equipped with a 488 nm laser and a pulsed White Light Laser (WLL) using 100× magnification. HepG2 (2×10^5 cells per well) were seeded in confocal dishes overnight. Then, cells were incubated with 500 nM of **Ly-Red-BODIPY** at 37 °C for 30 min, followed by three washes with PBS. Subsequently, the cells were incubated with **Gal_3-BODIPY** (500 nM), and then STED fluorescence imaging was conducted promptly; STED images were captured every 30 s for a duration of 5 min. BODIPY was excited at 488 nm using a white light laser, accepted at 500-550 nm and depleted with a 775 nm STED laser. **Ly-Red-BODIPY** excitation at 638 nm, emission at 640-700 nm and depletion with 775 nm STED laser. Image analysis was achieved using Leica Application Suite X (LAS X) and Fiji software.

S2. Additional figures

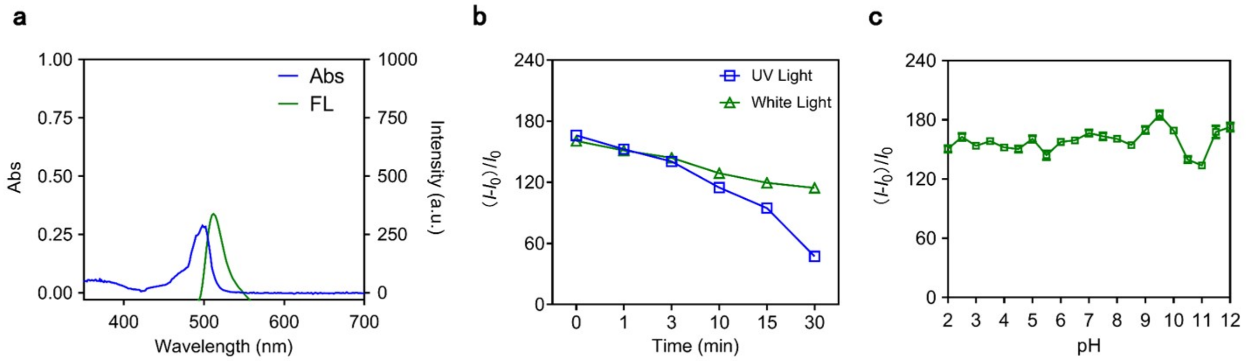

Figure S1. (a) Stacked absorption (40 μM) and fluorescence emission (10 μM, λ_{ex} = 488 nm) spectra of **Gal-BODIPY** measured in a PBS solution (0.01 M, pH 7.4) (b) Fluorescence changes of **Gal-BODIPY** (10 μM) in PBS (0.01 M, pH 7.4) under white light (560 nm, 1 W cm^{-2}) and UV light (365 nm, 1 W cm^{-2}) irradiation with time. (c) Fluorescence changes of **Gal-BODIPY** (5 μM) in PBS solutions with different pH (0.01 M, pH 2 - 12). The slit width was set at 5 nm.

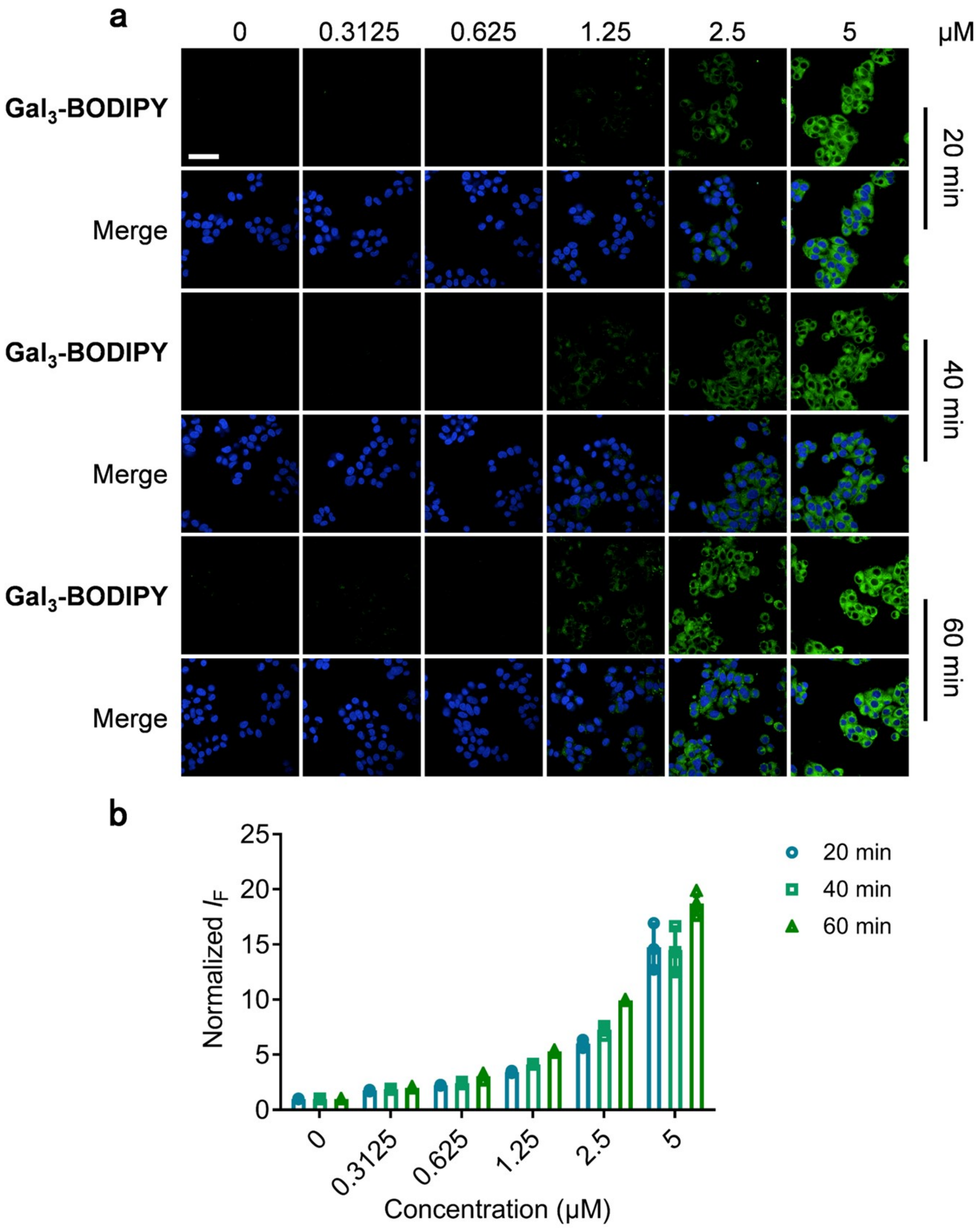

Figure S2. Concentration and time-dependent (a) fluorescence imaging and (b) quantification of HepG2 cells incubated with **Gal$_3$-BODIPY** (Scale bar = 100 μm).

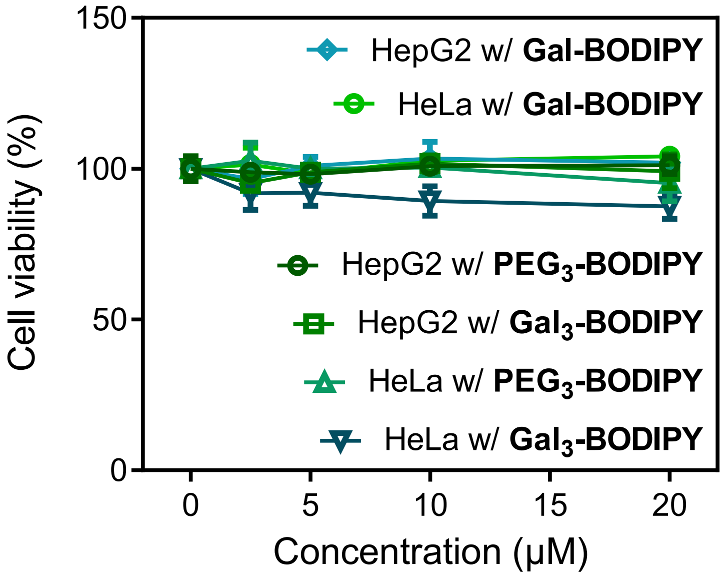

Figure S3. Viability of HepG2 and HeLa cells after incubation with different concentrations of **Gal$_3$-BODIPY**, **PEG$_3$-BODIPY** and **Gal-BODIPY** for 24 h.

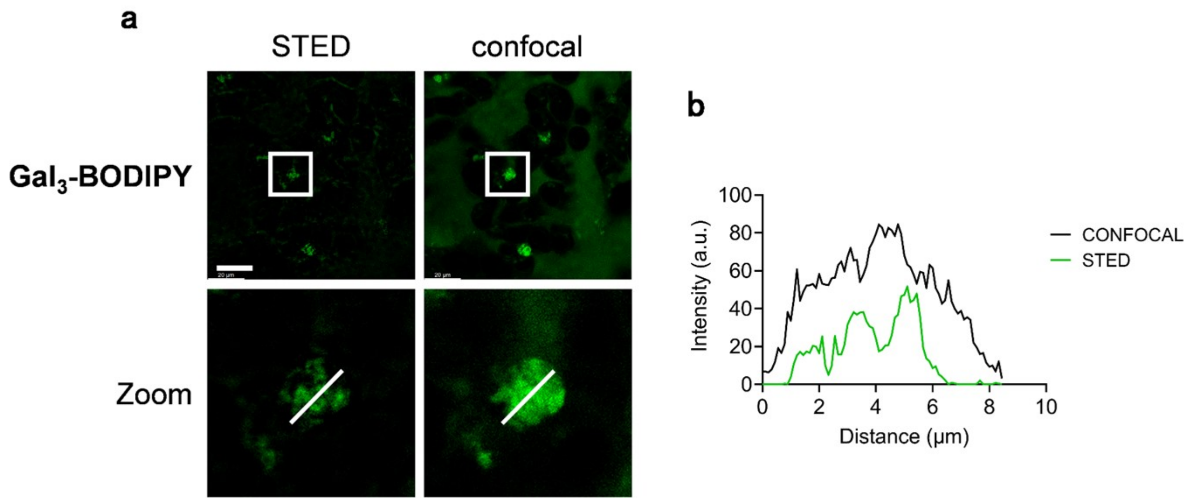

Figure S4. (a) Fluorescence imaging and (b) linear quantification of HepG2 cells incubated with **Gal$_3$-BODIPY** (10 μM) under STED mode and confocal mode (Scale bar = 20 μm).

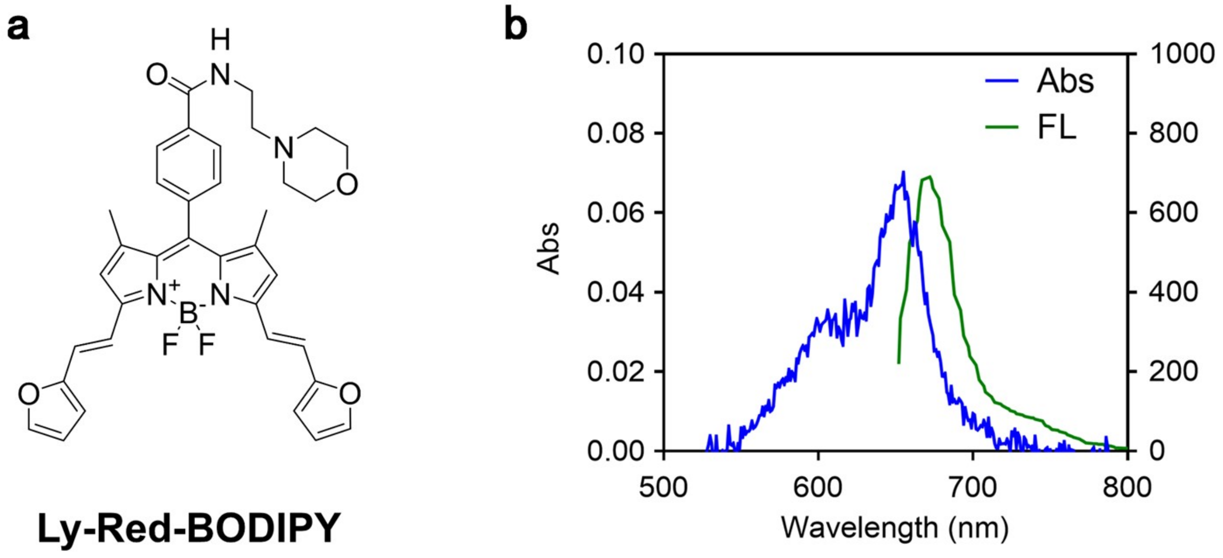

Figure S5. (a) Structure of **Ly-Red-BODIPY** and (b) stacked absorption (40 μM) and fluorescence emission spectra (10 μM) of **Ly-Red-BODIPY** measured in PBS (DMSO: PBS = 1:3 v/v). The excitation wavelength used for **Ly-Red-BODIPY** is 638 nm. The slit width was set at 5 nm.

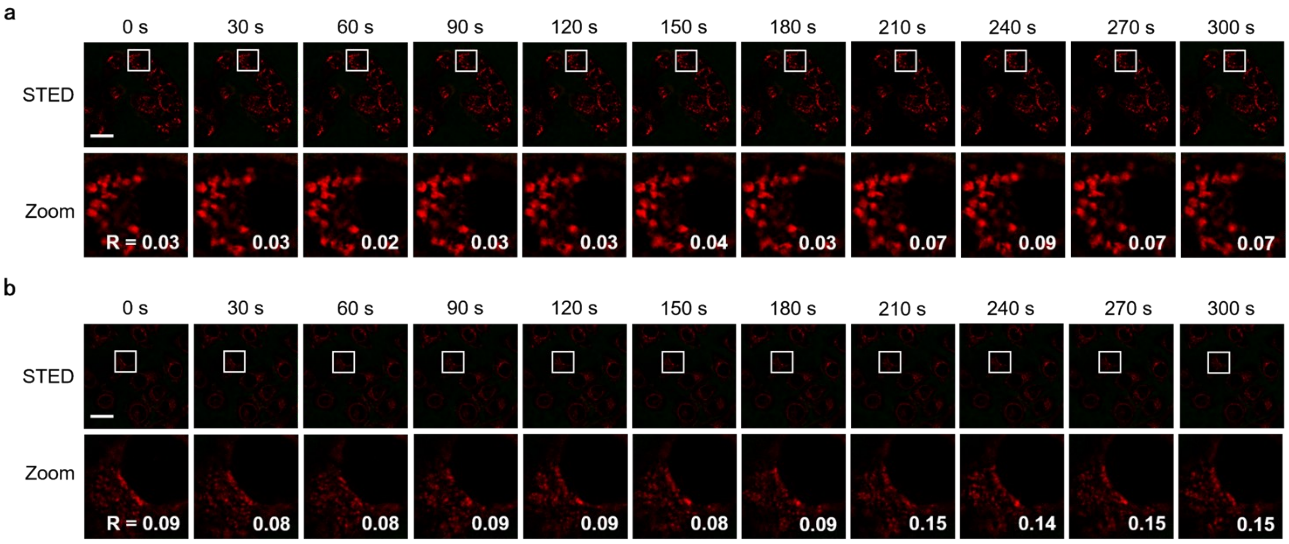

Figure S6. (a) Fluorescence imaging of HepG2 cells incubated with **PEG$_3$-BODIPY** and **Ly-Red-BODIPY** under STED mode. (b) Fluorescence imaging of HeLa cells incubated with **PEG$_3$-BODIPY** and **Ly-Red-BODIPY** under STED mode. BODIPY channel excitation at 488 nm, emission at 500-550 nm and depletion with 775 nm STED laser. **Ly-Red-BODIPY** excitation at 638 nm, emission at 640-700 nm and depletion with 775 nm STED laser. The corresponding Pearson's Correlation Coefficient is labelled in the right corner. (Scale bar = 25 μm).

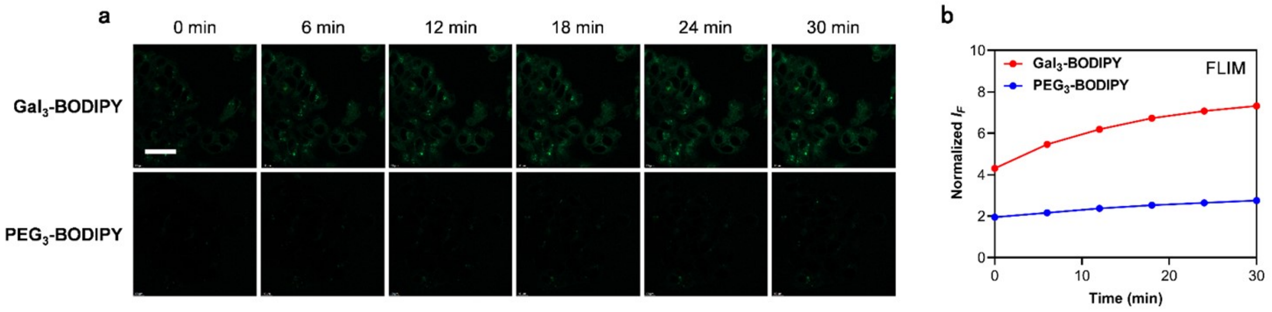

Figure S7. (a) Fluorescence imaging and (b) quantification of HepG2 cells incubated with **Gal$_3$-BODIPY** or **PEG$_3$-BODIPY** under FLIM mode (Scale bar = 25 μm).

Table S1. Photophysical properties of **Gal_3-BODIPY**

Solvent	ε (10^5 M^{-1} cm^{-1})	λ_{abs} (nm)	λ_{em} (nm)	Stokes shift (cm^{-1})	Φ_F	τ (ns)
PBS	3.3	502	516	540	0.49	1.42

S3. Original spectra of new compounds

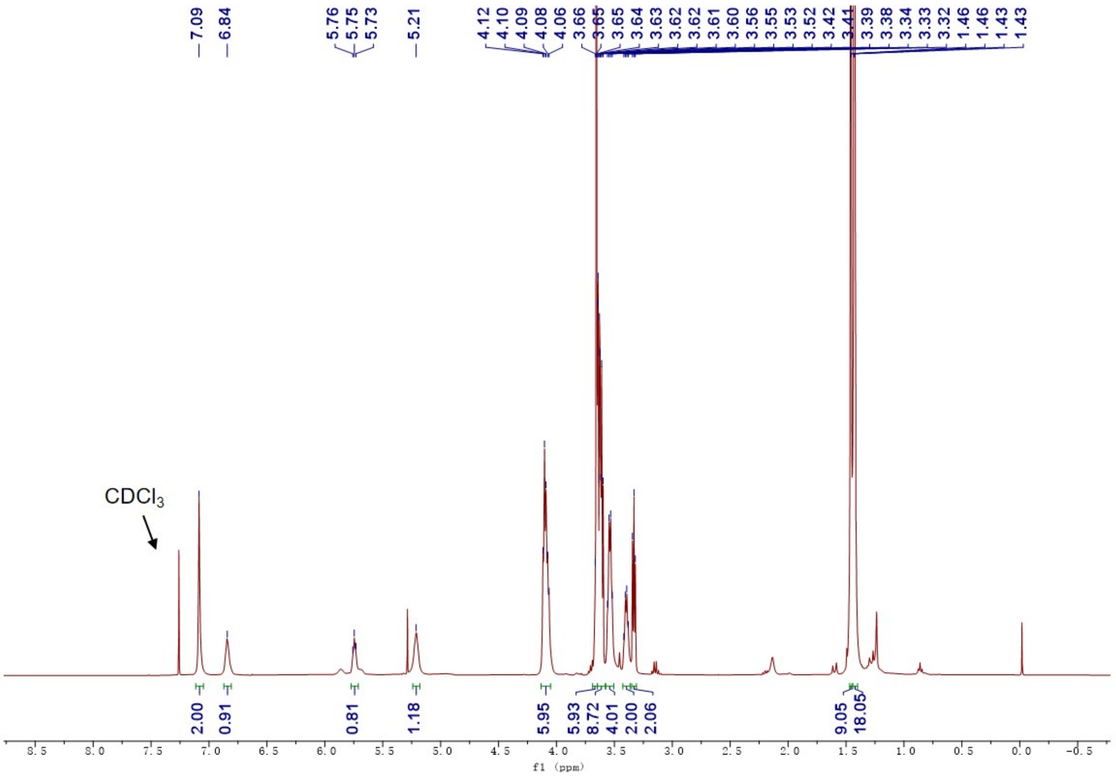

Figure S8. ^{1}H NMR spectrum of **3**.

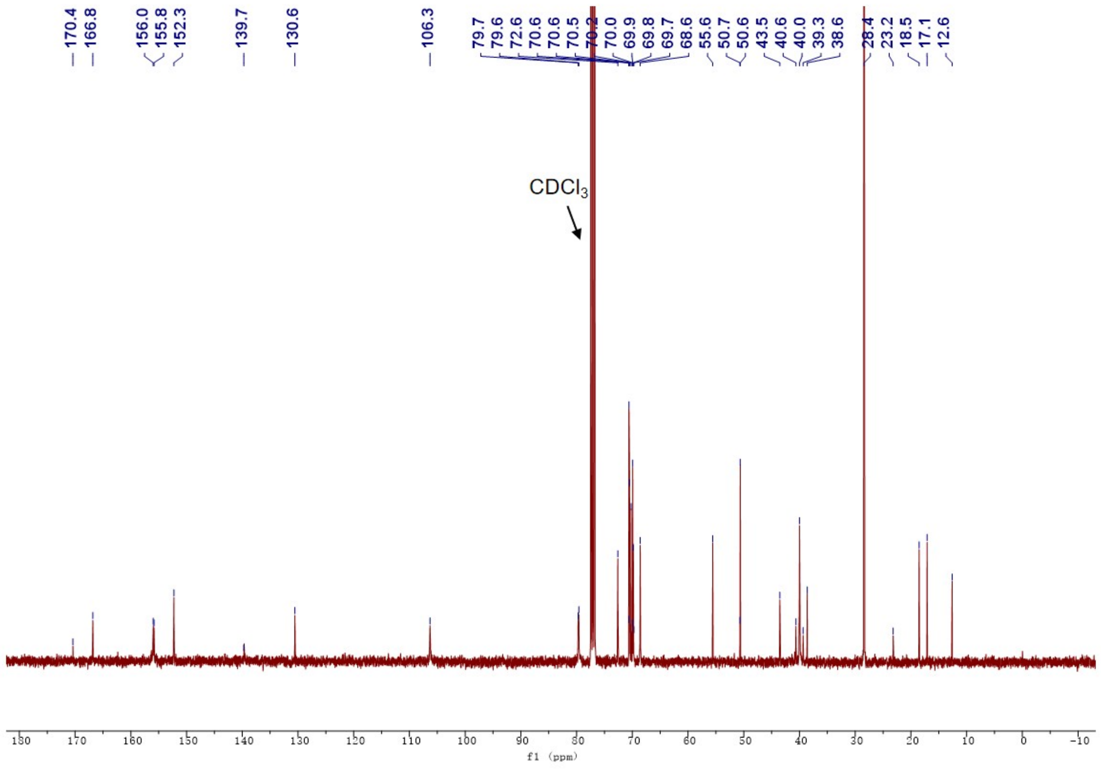

Figure S9 ^{13}C NMR spectrum of **3**.

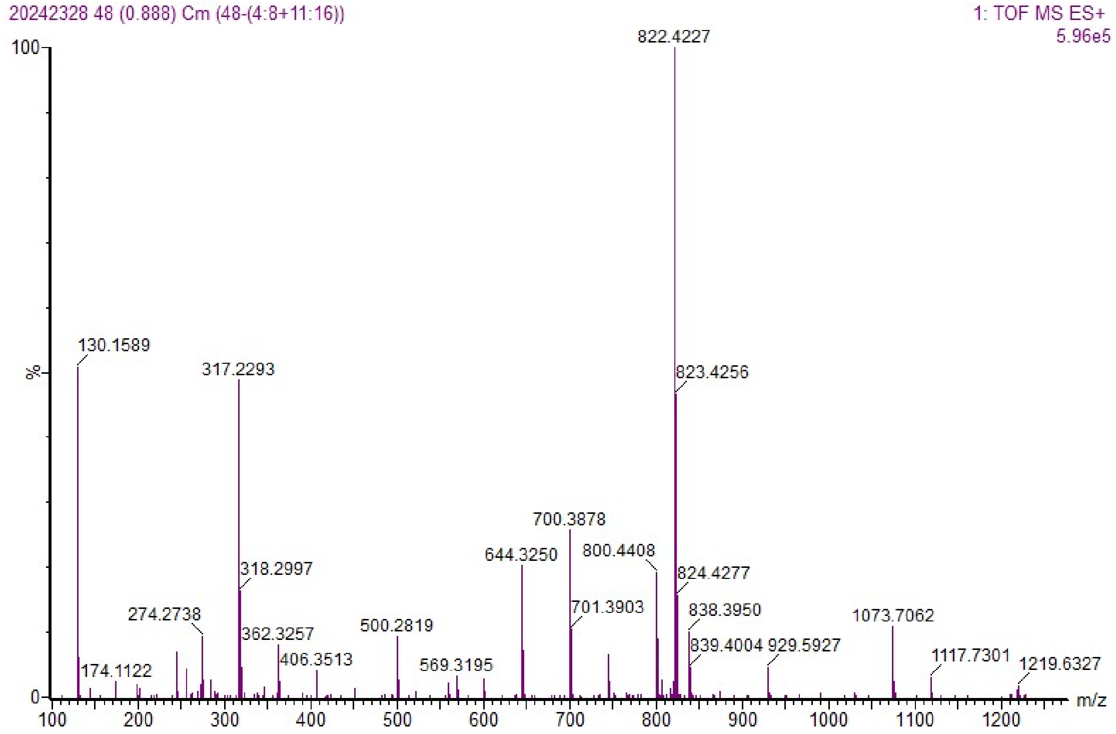

Figure S10. ESI-TOF spectrum of **3**.

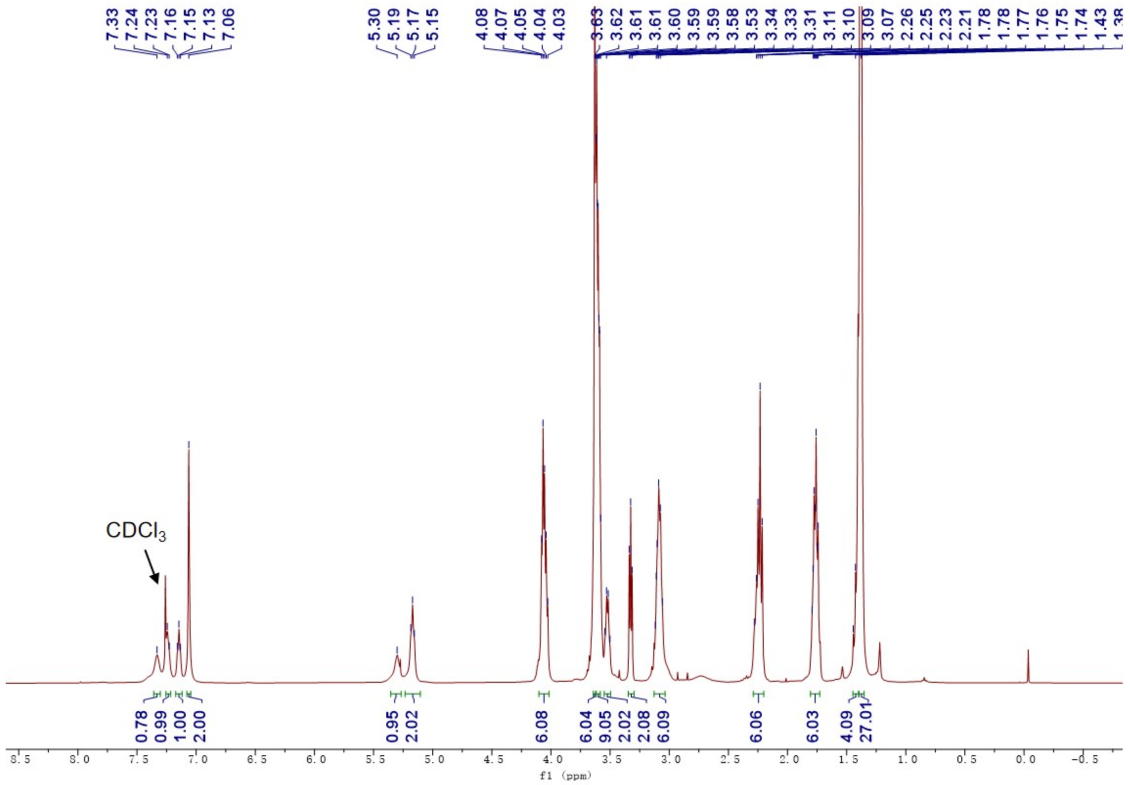

Figure S11. ^{1}H NMR spectrum of **4**.

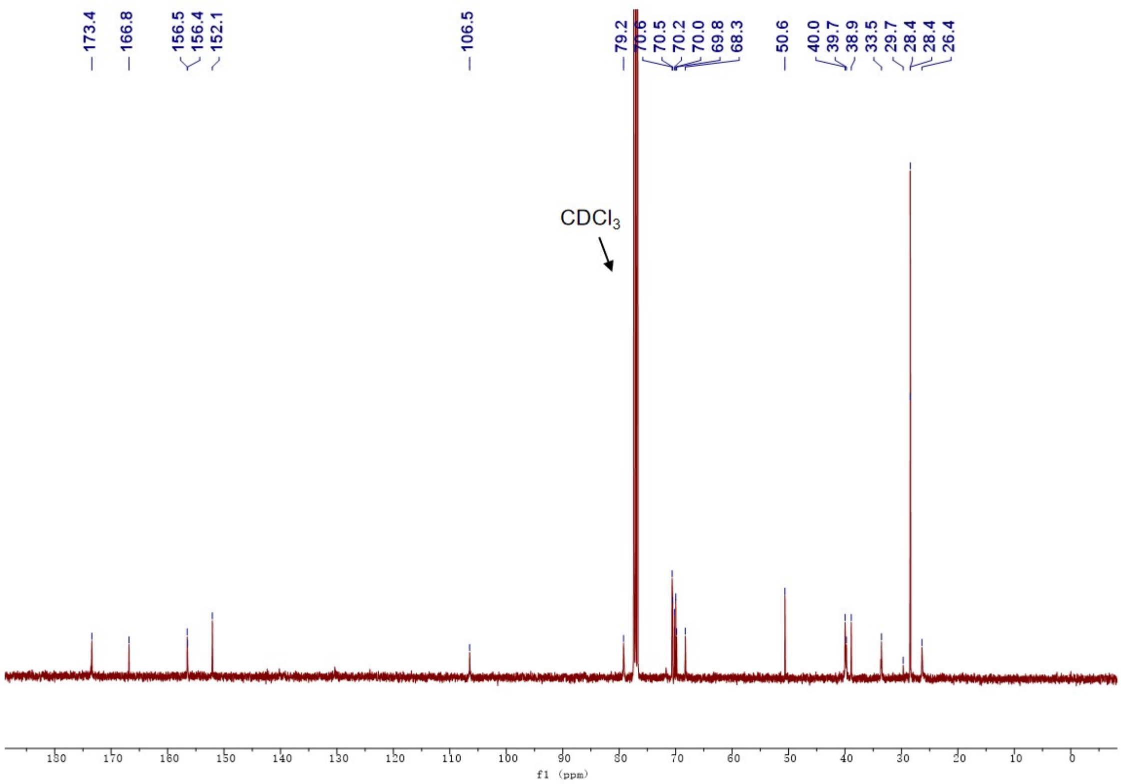

Figure S12. ^{13}C NMR spectrum of **4**.

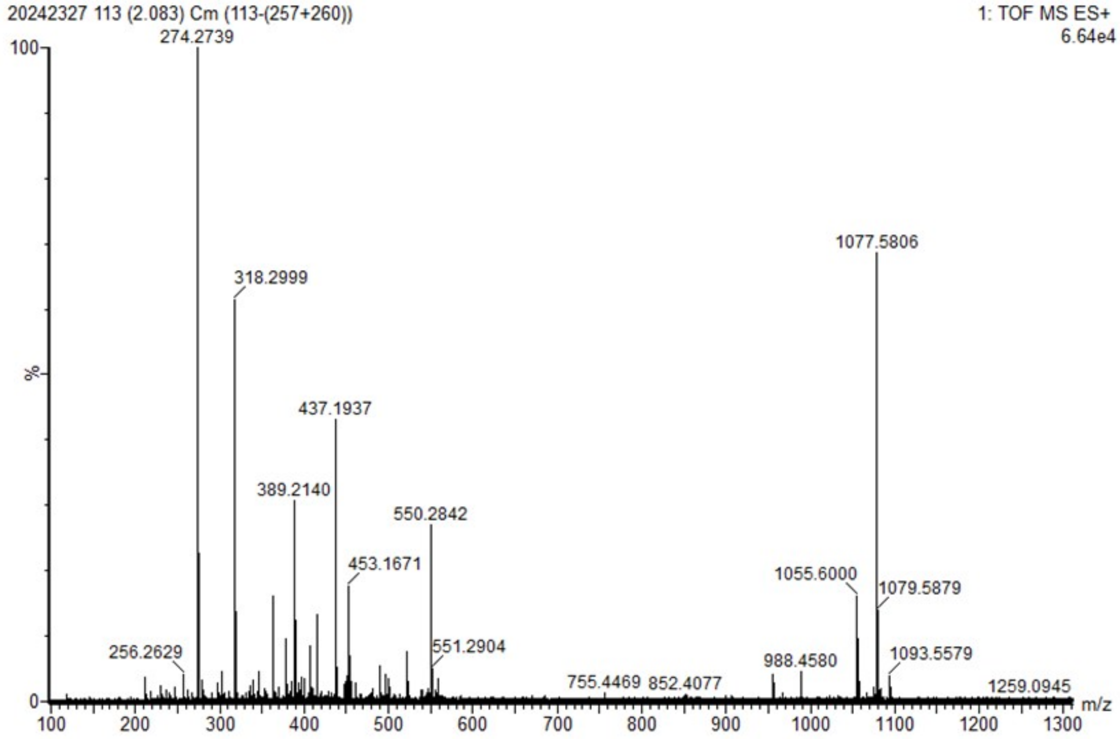

Figure S13. ESI-TOF spectrum of **4**.

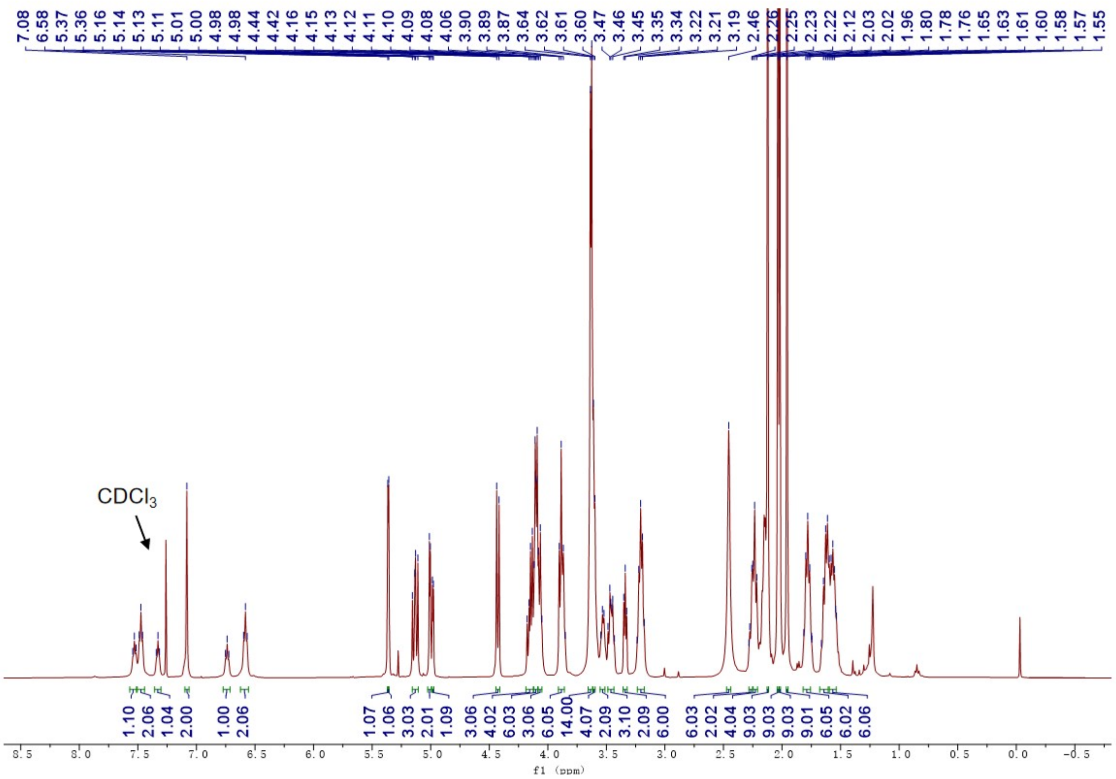

Figure S14. ^{1}H NMR spectrum of **5**.

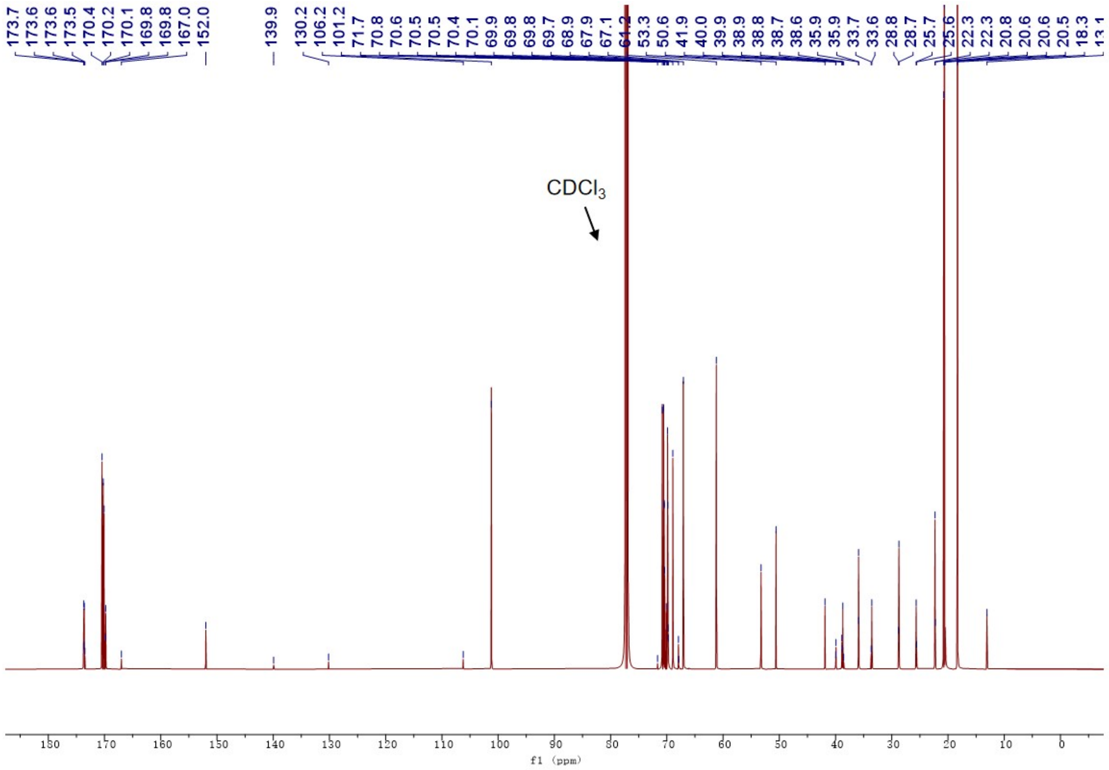

Figure S15. ^{13}C NMR spectrum of **5**.

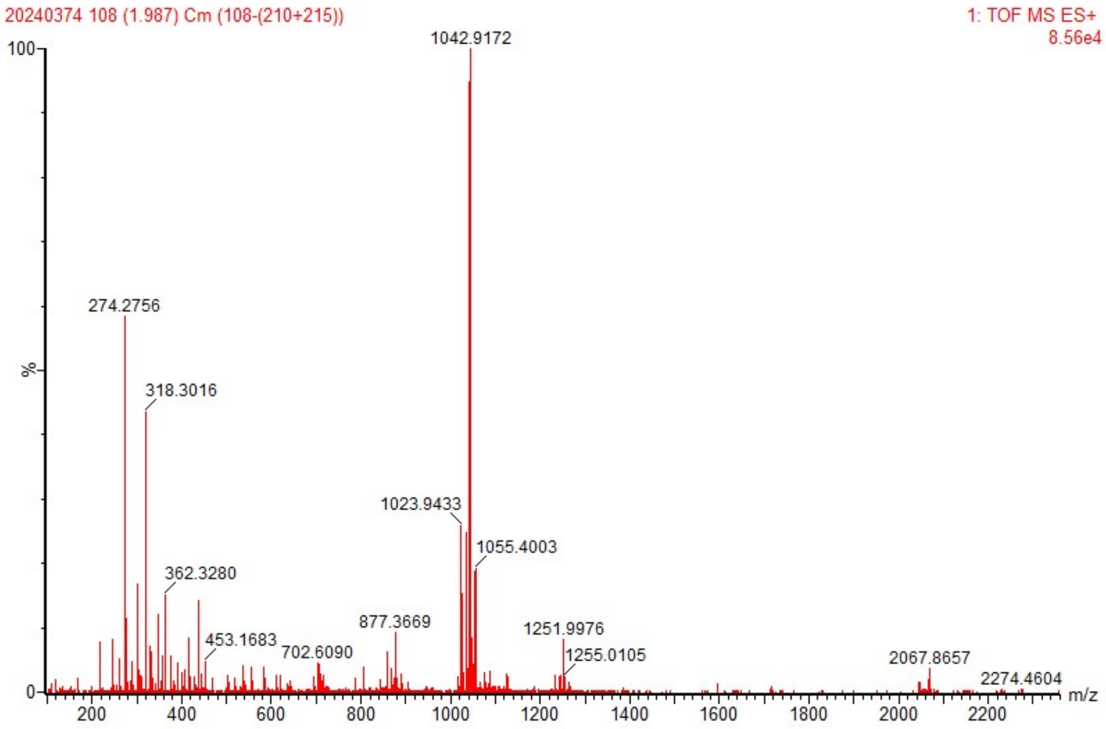

Figure S16. ESI-TOF spectrum of **5**.

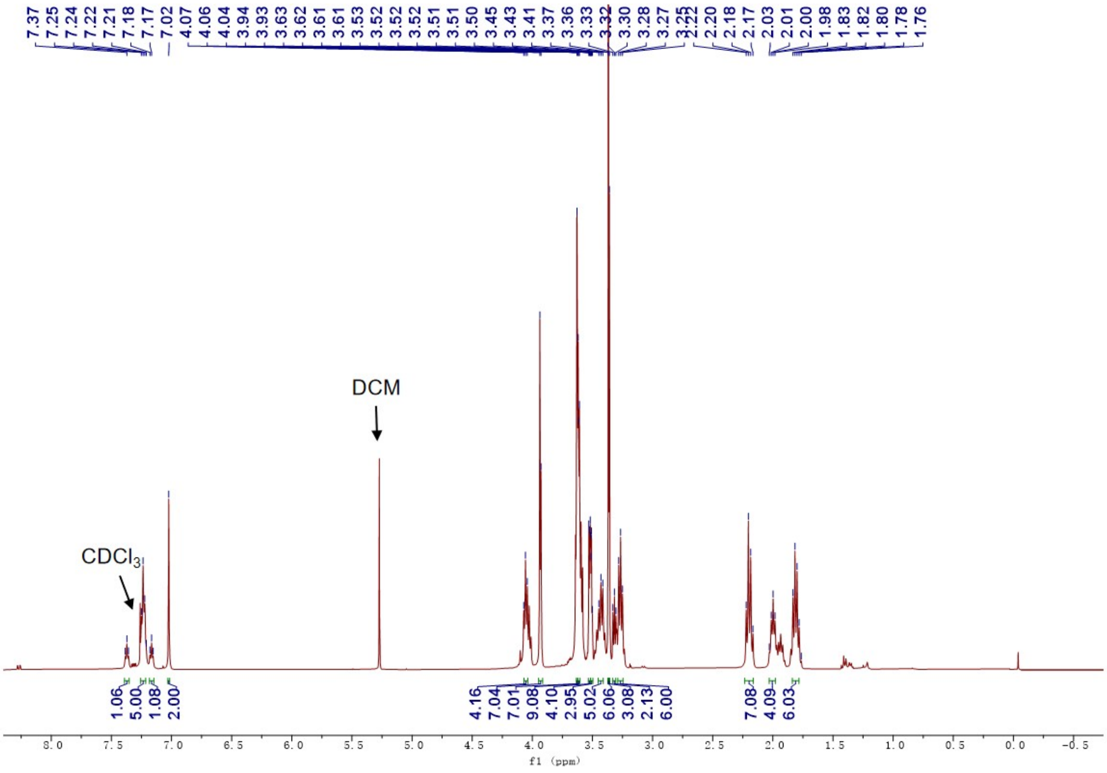

Figure S17. ^{1}H NMR spectrum of **6**.

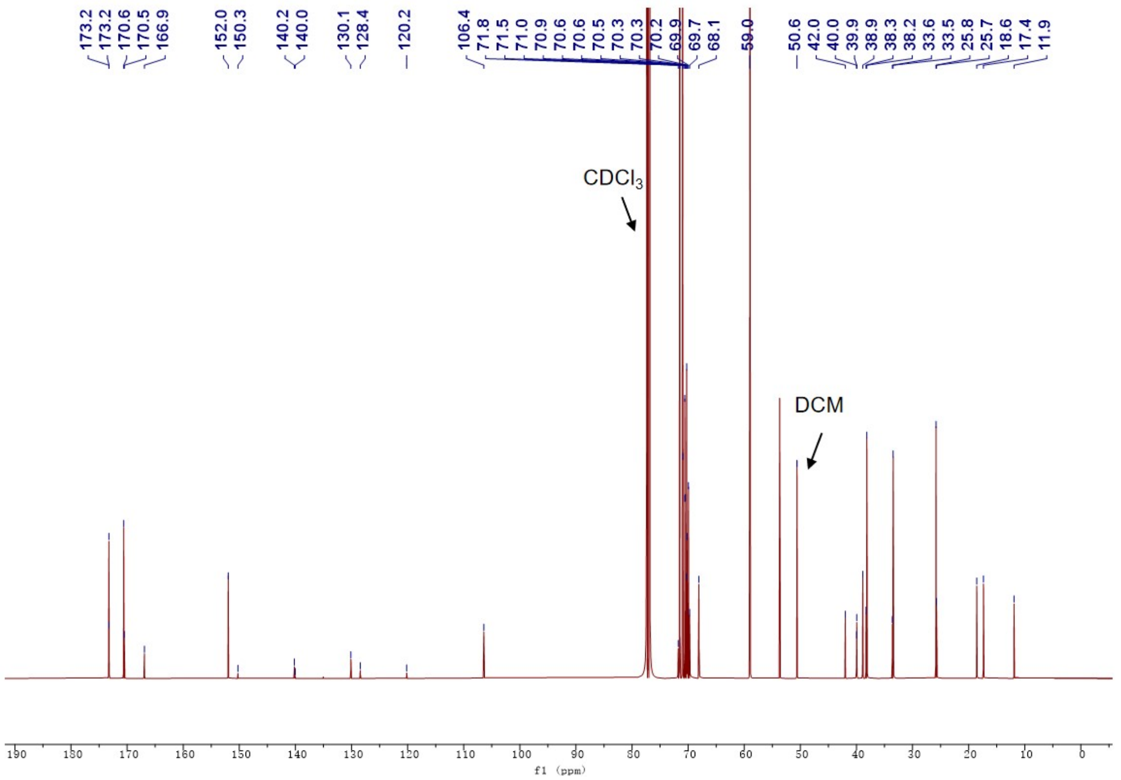

Figure S18. ^{13}C NMR spectrum of **6**.

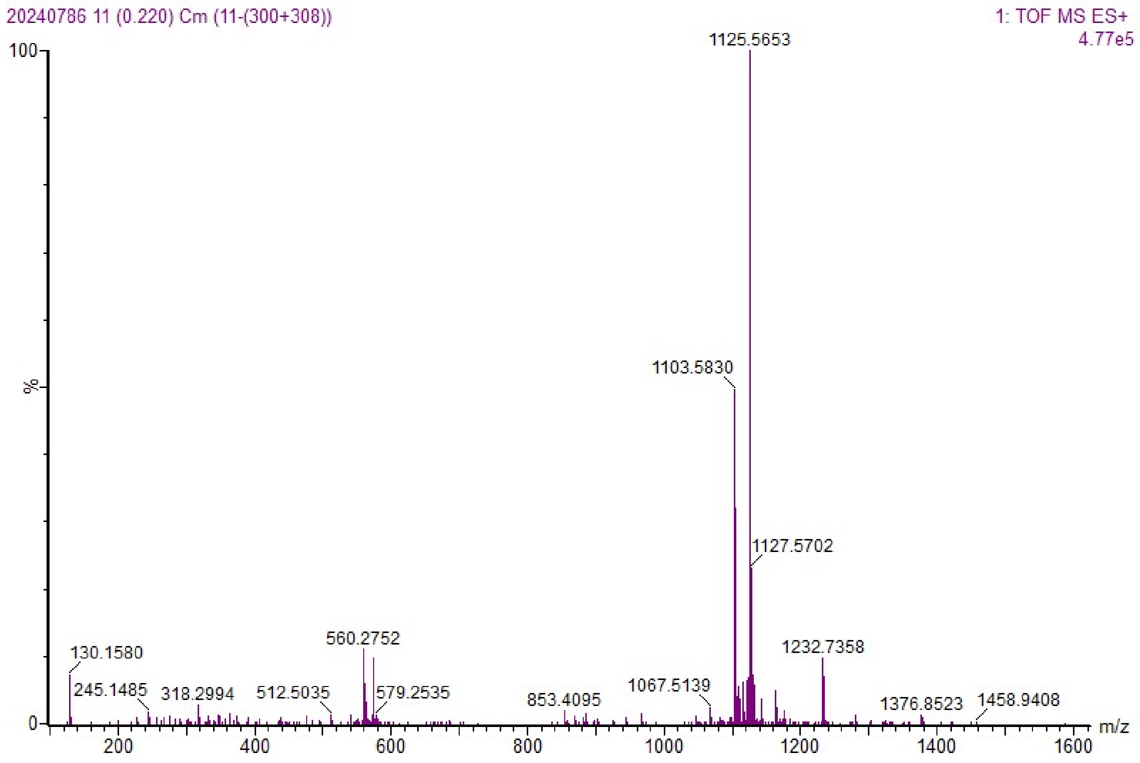

Figure S19. ESI-TOF spectrum of **6**.

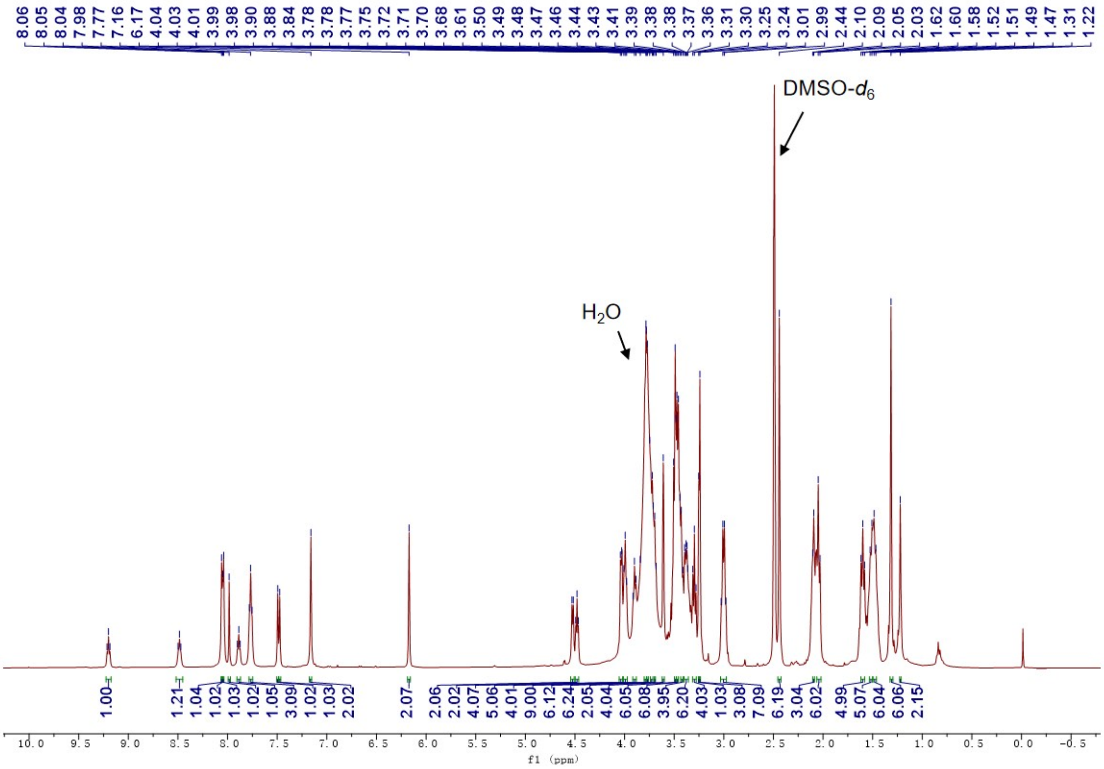

Figure S20. ^{1}H NMR spectrum of **Gal$_3$-BODIPY**.

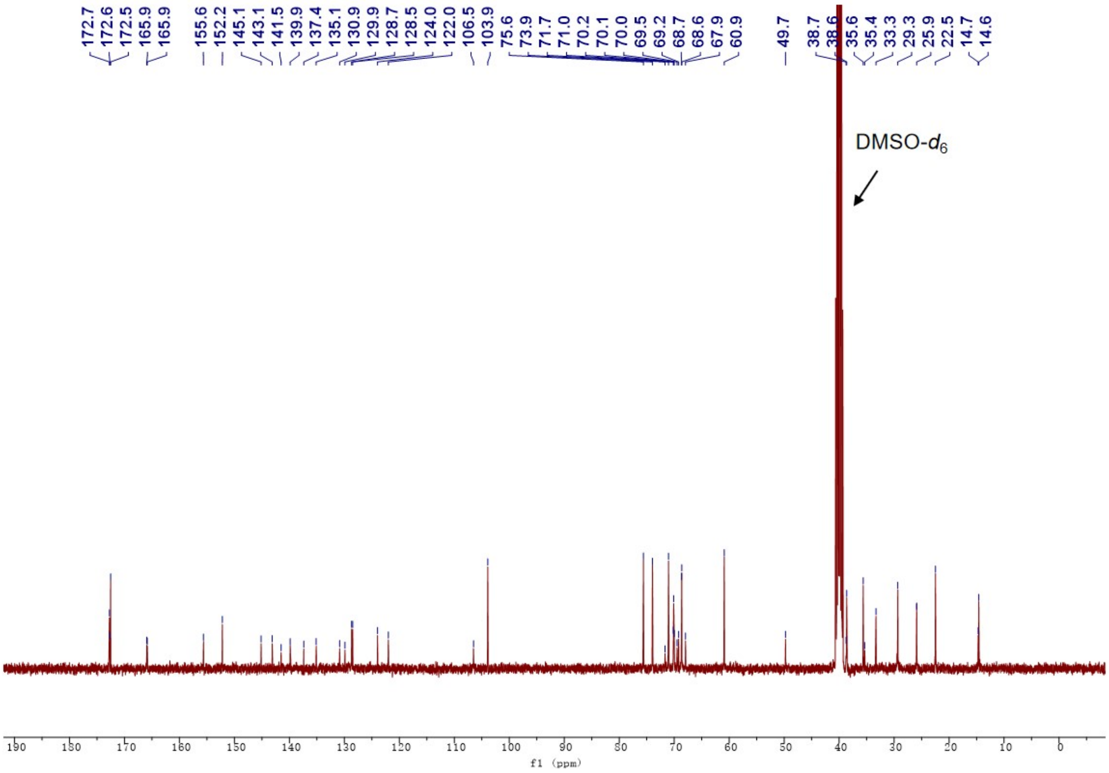

Figure S21. ^{13}C NMR spectrum of **Gal$_3$-BODIPY**.

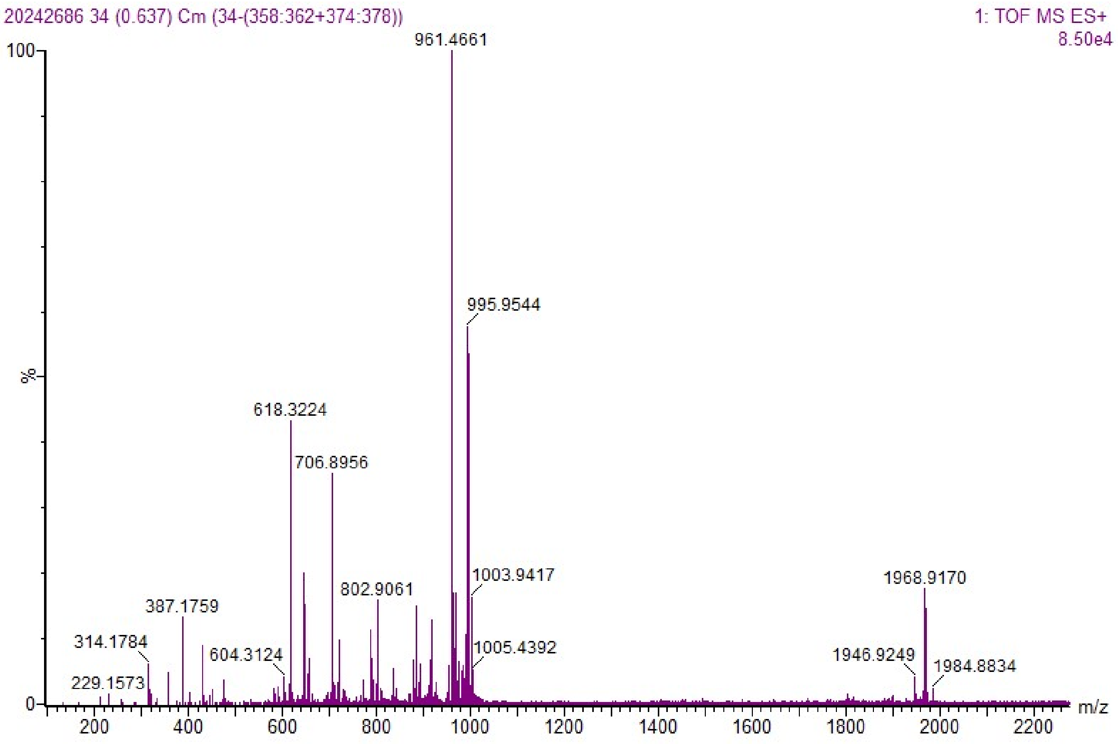

Figure S22. ESI-TOF Spectrum of **Gal$_3$-BODIPY**.

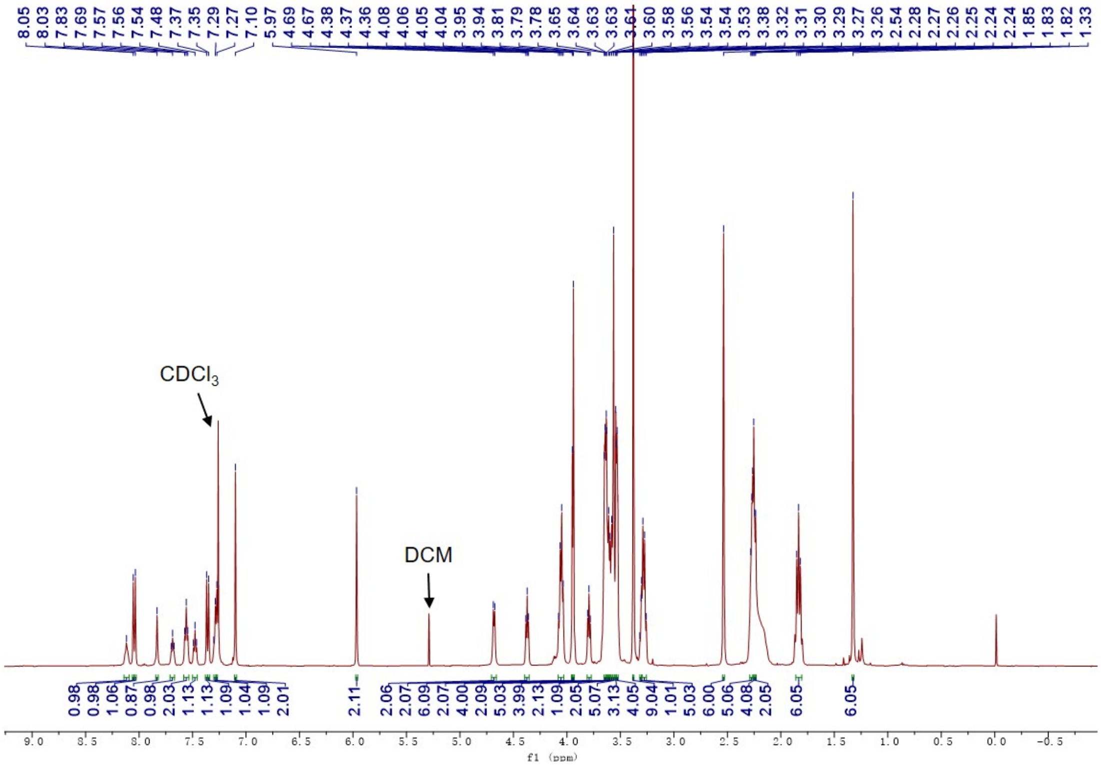

Figure S23. ^{1}H NMR spectrum of **PEG$_3$-BODIPY**.

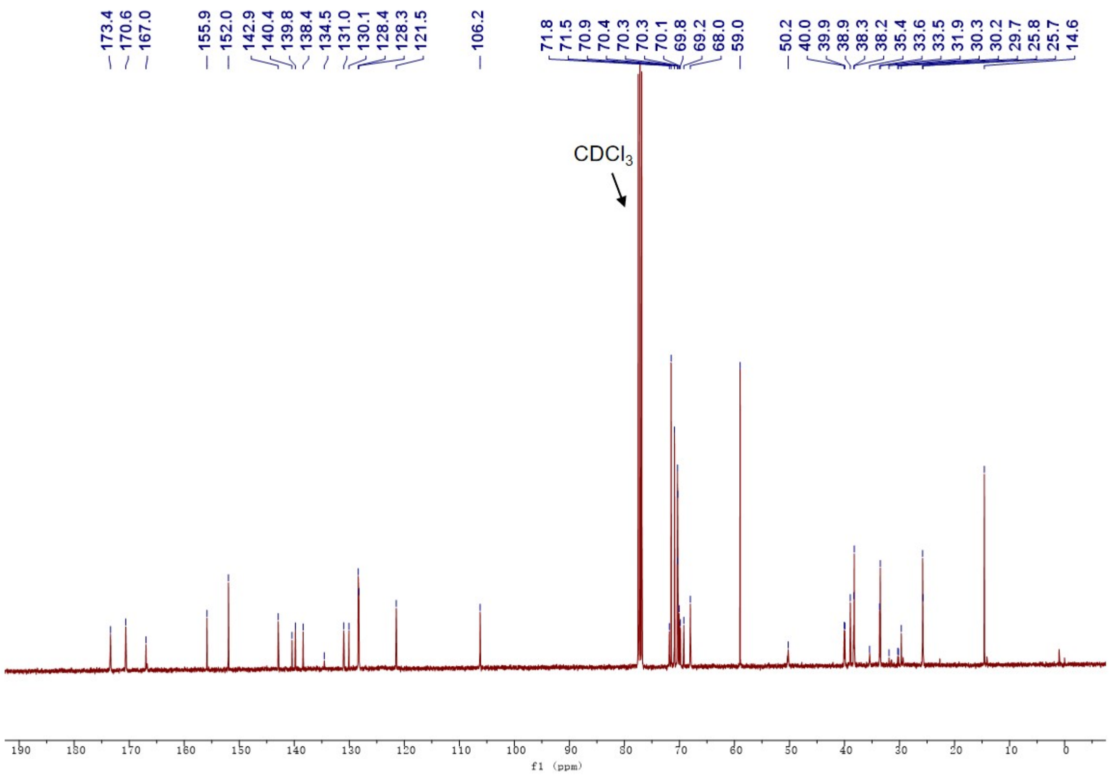

Figure S24. ^{13}C NMR spectrum of **PEG$_3$-BODIPY**.

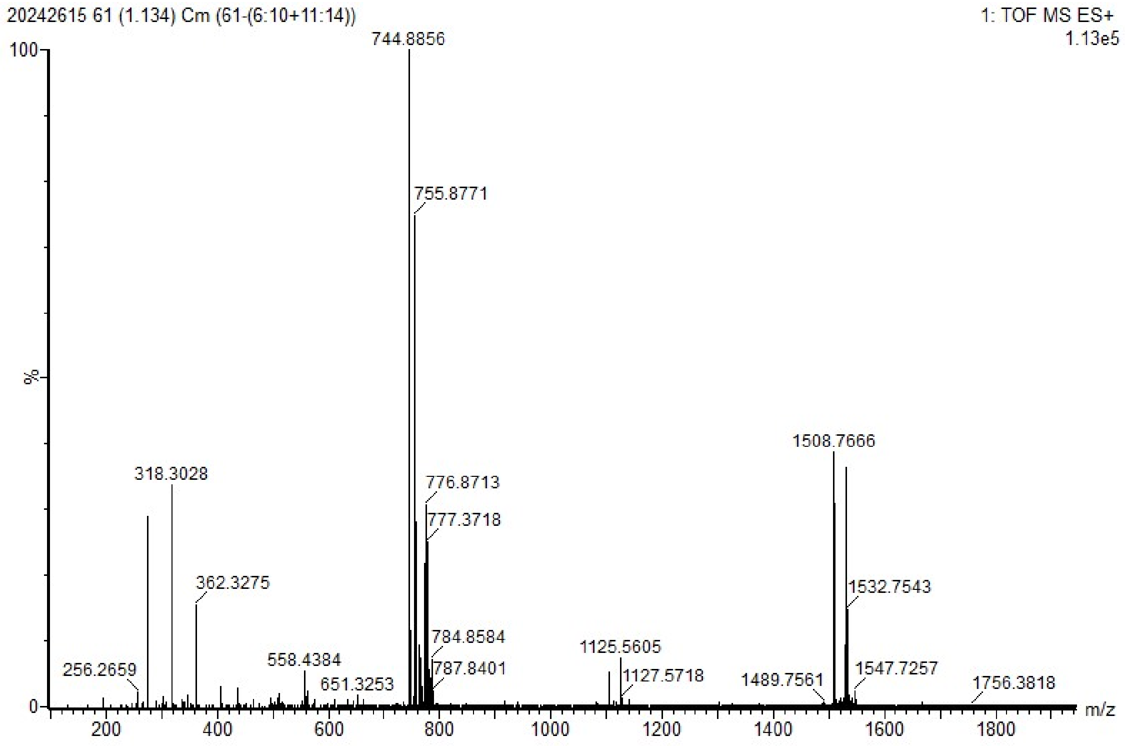

Figure S25. ESI-TOF spectrum of **PEG$_3$-BODIPY**.

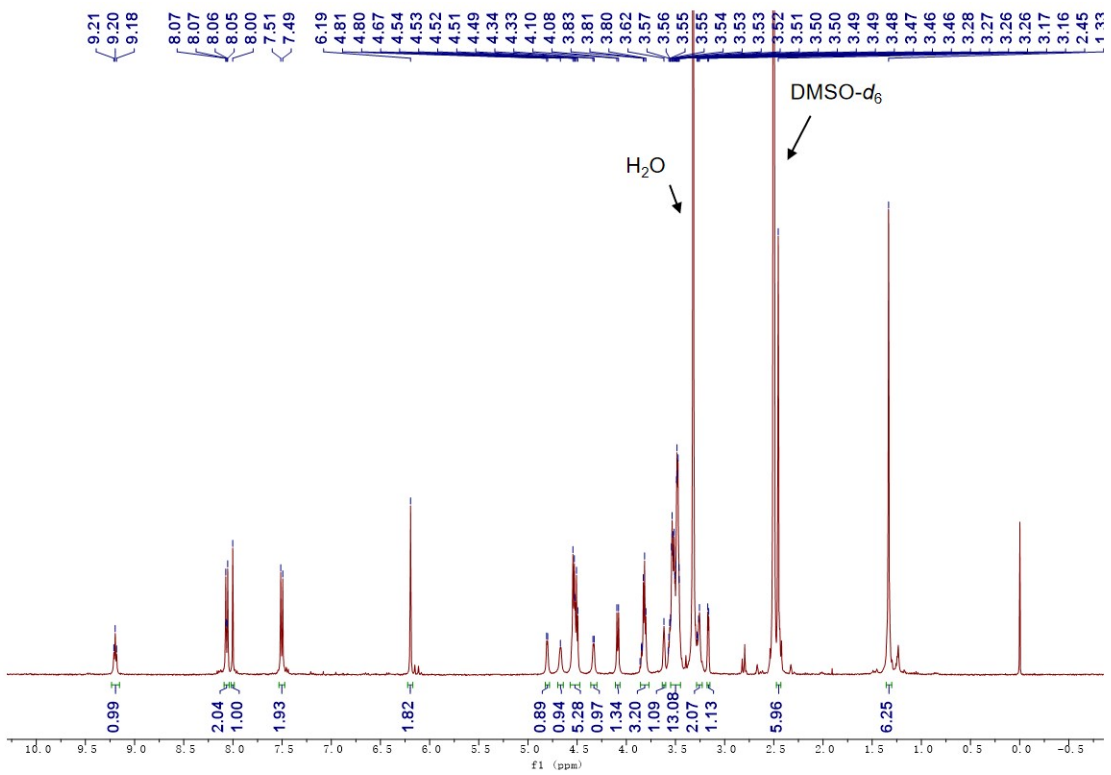

Figure S26. ^{1}H NMR spectrum of **Gal-BODIPY**.

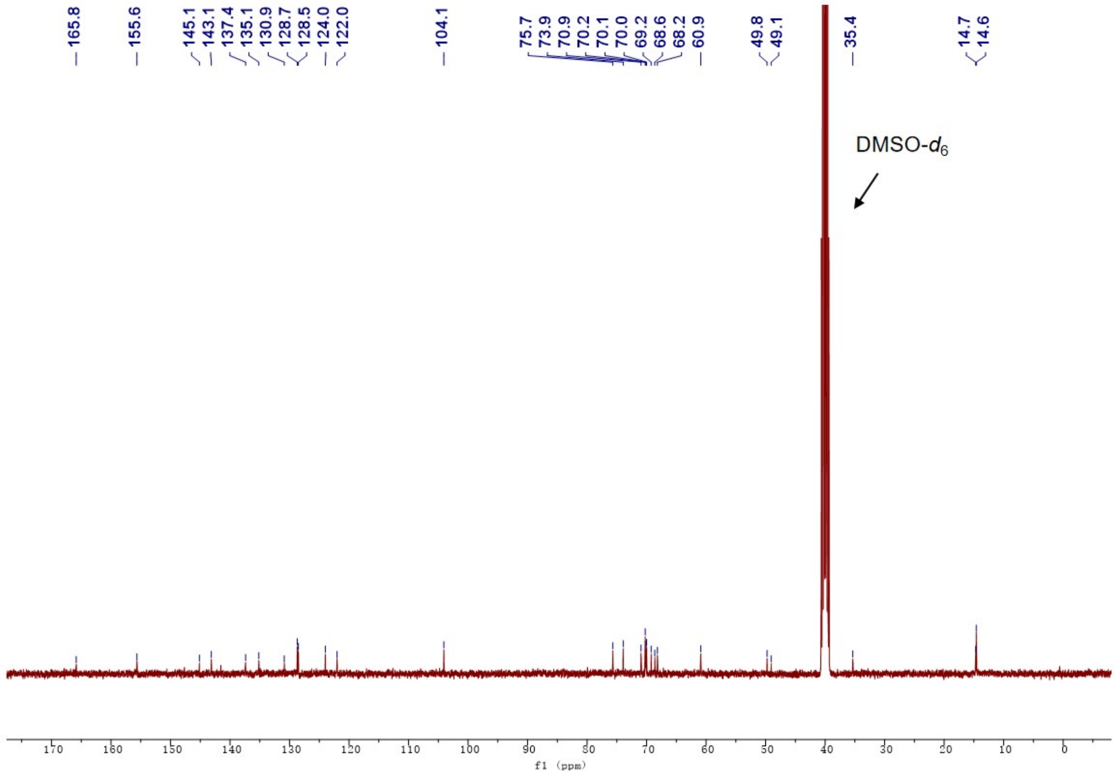

Figure S27. ^{13}C NMR spectrum of **Gal-BODIPY**.

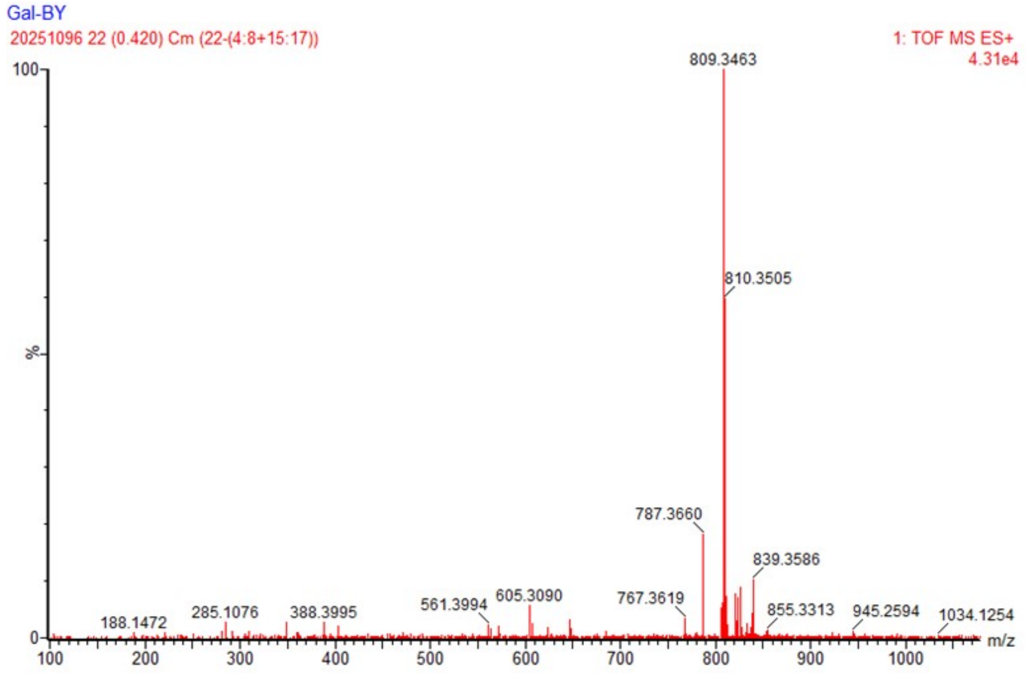

Figure S28. ESI-TOF spectrum of **Gal-BODIPY**.

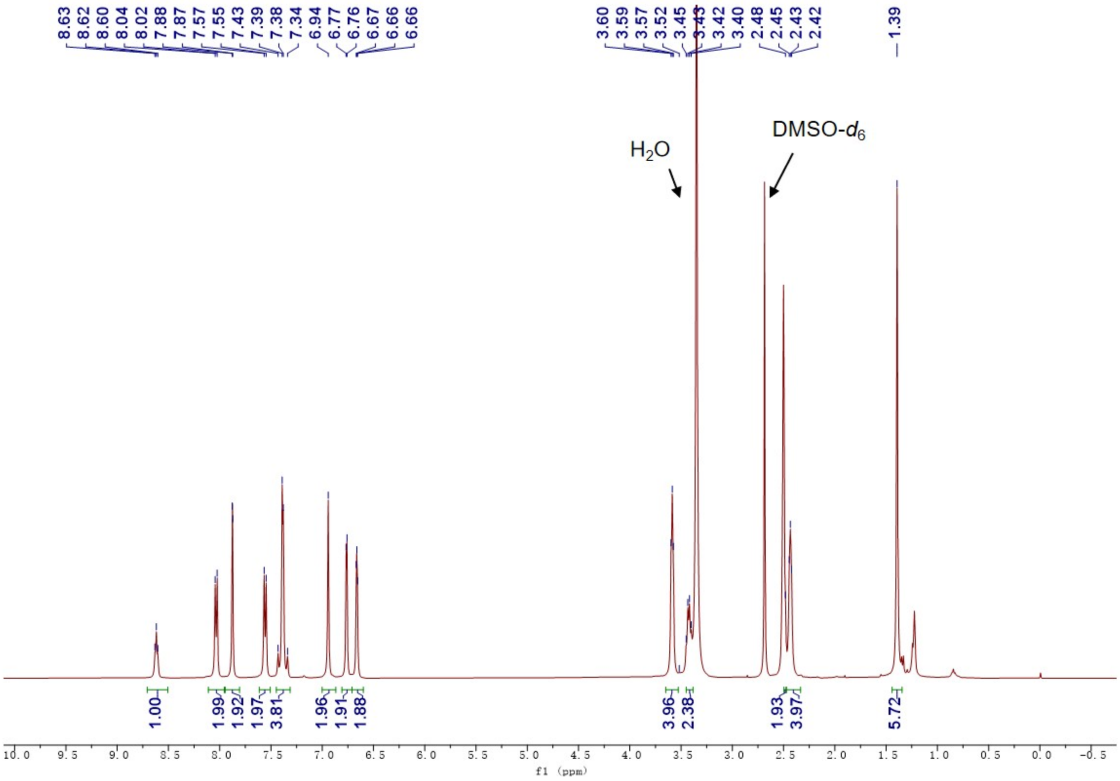

Figure S29. ^{1}H NMR spectrum of **Ly-Red-BODIPY**.

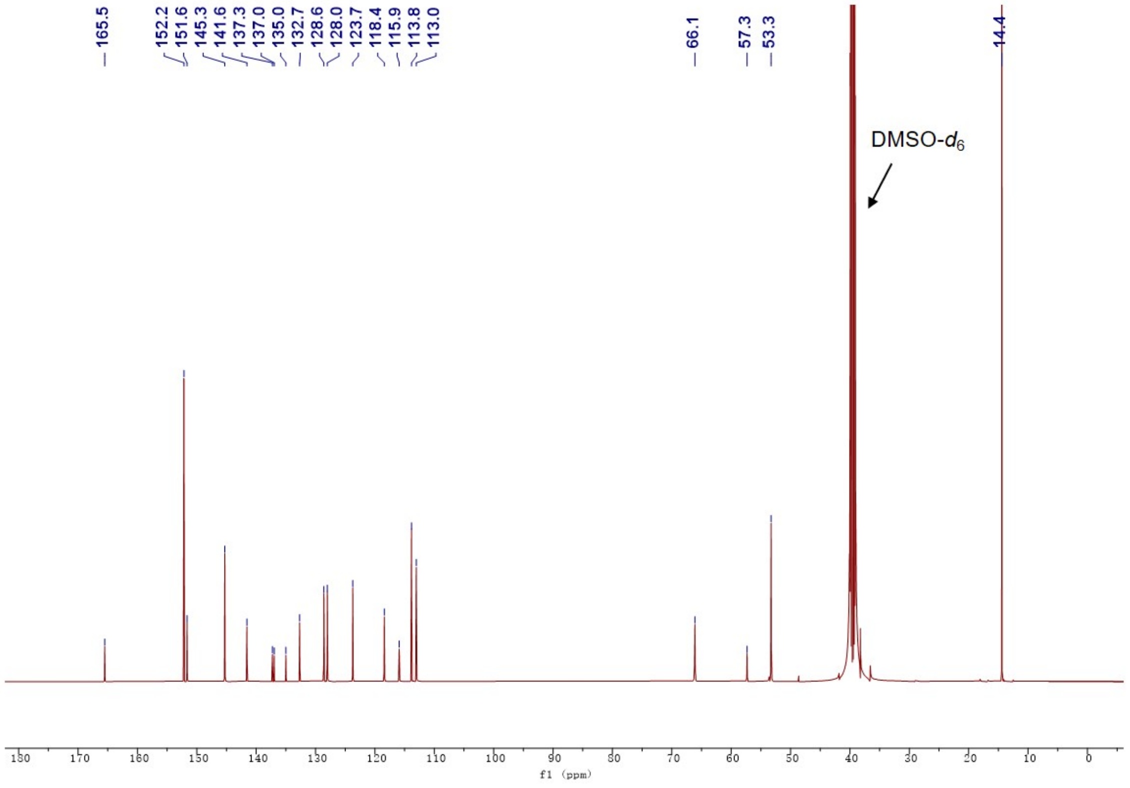

Figure S30. ^{13}C NMR spectrum of **Ly-Red-BODIPY**.

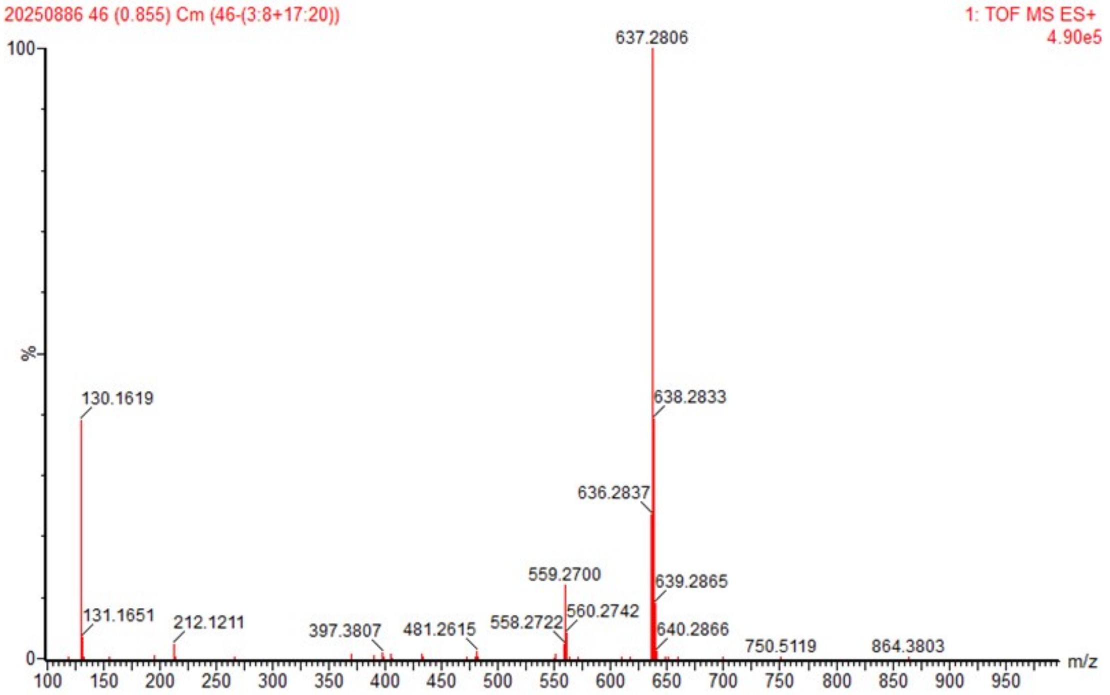

Figure S31. ESI-TOF Spectrum of **Ly-Red-BODIPY**.

S4. Additional references

[1] C. Quintela, F. G.-D. Portillo and A. San-Félix, *Molecules*, 2018, **23**, 1513.

[2] B.-L. Zhang, W. Wang, US Pat., US2020369703A1, 2020.

[3] C. Zhao, A. Fernandez, N. Avlonitis, G. V. Velde, M. Bradley, N. D. Read and M. Vendrell, *ACS Comb. Sci.*, 2016, **18**, 689–696.

[4] W.-W. Haoyang, Z.-T. Li and J.-L. Hou, *Chem. Comm.*, 2021, **57**, 1097–1100.

[5] S. Ansteatt, M. Ptaszek and A. Meares, *J. Org. Chem.*, 2021, **86**, 8755–8765

[6] C. Würth, M. Grabolle, J. Pauli, M. Spieles and U. Resch-Genger, *Nat. Protoc.*, 2013, **8**, 1535–1550.
